# Extracellular DNAses Facilitate Antagonism and Coexistence in Bacterial Competitor-Sensing Interference Competition

**DOI:** 10.1128/aem.01437-22

**Published:** 2022-11-14

**Authors:** Aoi Ogawa, Christophe Golé, Maria Bermudez, Odrine Habarugira, Gabrielle Joslin, Taylor McCain, Autumn Mineo, Jennifer Wise, Julie Xiong, Katherine Yan, Jan A. C. Vriezen

**Affiliations:** a Department of Biological Sciences, Smith College, Northampton, Massachusetts, USA; b Department of Mathematical Sciences, Smith College, Northampton, Massachusetts, USA; c Department of Statistics and Data Sciences, Smith College, Northampton, Massachusetts, USA; University of Illinois at Urbana-Champaign

**Keywords:** competition models, exDNases, cellular automaton, coexistence, secondary metabolites, antibiotics, soil, antagonism, soil microbiology

## Abstract

Over the last 4 decades, the rate of discovery of novel antibiotics has decreased drastically, ending the era of fortuitous antibiotic discovery. A better understanding of the biology of bacteriogenic toxins potentially helps to prospect for new antibiotics. To initiate this line of research, we quantified antagonists from two different sites at two different depths of soil and found the relative number of antagonists to correlate with the bacterial load and carbon-to-nitrogen (C/N) ratio of the soil. Consecutive studies show the importance of antagonist interactions between soil isolates and the lack of a predicted role for nutrient availability and, therefore, support an *in situ* role in offense for the production of toxins in environments of high bacterial loads. In addition, the production of extracellular DNAses (exDNases) and the ability to antagonize correlate strongly. Using an *in domum*-developed probabilistic cellular automaton model, we studied the consequences of exDNase production for both coexistence and diversity within a dynamic equilibrium. Our model demonstrates that exDNase-producing isolates involved in amensal interactions act to stabilize a community, leading to increased coexistence within a competitor-sensing interference competition environment. Our results signify that the environmental and biological cues that control natural-product formation are important for understanding antagonism and community dynamics, structure, and function, permitting the development of directed searches and the use of these insights for drug discovery.

**IMPORTANCE** Ever since the first observation of antagonism by microorganisms by Ernest Duchesne (E. Duchesne, Contribution à l’étude de la concurrence vitale chez les microorganisms. Antagonism entre les moisissures et les microbes, These pour obtenir le grade de docteur en medicine, Lyon, France, 1897), many scientists successfully identified and applied bacteriogenic bioactive compounds from soils to cure infection. Unfortunately, overuse of antibiotics and the emergence of clinical antibiotic resistance, combined with a lack of discovery, have hampered our ability to combat infections. A deeper understanding of the biology of toxins and the cues leading to their production may elevate the success rate of the much-needed discovery of novel antibiotics. We initiated this line of research and discovered that bacterial reciprocal antagonism is associated with exDNase production in isolates from environments with high bacterial loads, while diversity may increase in environments of lower bacterial loads.

## INTRODUCTION

The evolutionary arms race of effective antimicrobial development and subsequent development of antibiotic resistance in microbes has become a hindrance in our pursuit of new and effective antimicrobials (1). In a commentary in *Nature Microbiology*, Kolter and van Wezel (2) argued that the era of novel antibiotic discovery via brute force has ended. New approaches are needed to sample the underexploited niches in environments like soil, e.g., those developed by Ling et al. (3). In addition, approaches based on biological and ecological insights may lead us to antagonist bacteria with a reduced probability of rediscovering what is already known. Due to their disputed roles (4), many of these insights remain unknown. However, in recent years, several studies have addressed polymicrobial communities from different environments to further our understanding of dynamic interactions between bacteria and the consequences for diversity. Interactions can be cooperative, neutral, or antagonist, unidirectional or reciprocal, symmetrical or asymmetrical, social or asocial, transitive or nontransitive, sympatric or allopatric, structured or unstructured and are used in (mathematical) models to predict structure, stability, and diversity (5–15). Although varied in their specifics, all studies acknowledge inherent problems in testing that are hard to solve. For example, the culture dependence of the bacterial isolates may not be representative of *in situ* populations. Furthermore, the choice of media, nutrient availability, experimental design, and source of isolates vary between studies, resulting in often mixed or highly selective populations and making it difficult to directly compare results (5, 7, 11, 14). Studies attempting to more deeply understand the consequences of interactions within populations of antagonists are scarce, e.g., social versus asocial, sympatric and allopatric, the medium used, or *in vitro* versus *in vivo* studies (5, 8, 9, 11); however, comparative studies between populations are limited, and further exploration is warranted.

Although the role of bacteriogenic toxins in their environment is still up for debate, the anthropocentric point of view is that bioactive compounds are produced as offensive mechanisms against competitor bacteria. However, secreted bioactive compounds undergo diffusion and the resulting ambient concentrations are too low to inhibit growth on realistic length and time scales (16), while hormesis affects phenotypes (17). Regardless of the true *in situ* role, toxins that kill or inhibit growth at high concentrations lead to negative consequences to neighboring cells when in close proximity to antagonists (18, 19). Therefore, we hypothesize that an increased bacterial load will lead to greater benefits for the antagonists, resulting in an elevated presence of said antagonists. Additionally, recent work showed that interactions are overrepresented intergenerically and are inversely related to phylogenetic, metabolic, and functional distance (7, 11, 14). As a result, with an increasing bacterial load, one expects a decrease in the diversity of antagonists and a decrease in the ability to find novelty. These arguments echo the theoretical considerations published by Curtis and Sloan (20).

Various bacterial competition models exist; however, in this paper, we will focus on the following two models: exploitation and interference competition (21). Exploitation competition is practiced by bacteria able to efficiently use resources, while interference competition is practiced by those that produce toxins to ward off competitors. Russel et al. (11) studied the trade-off between exploitation competition and interference competition and found that antagonists that practice interference have a wider metabolic-niche space and a larger network. Within the general theoretical realm of interference competition, the cue for antagonism is either environmental or competitive. Theoretically, it is possible to distinguish between competitor-sensing interference competition (CSIC model) and nutrient deprivation-sensing interference competition (NDSIC model). In the case of the former, microbial populations in environments with higher bacterial loads are expected to have a higher connectance (10) than populations from environments with low bacterial loads. In contrast, in the latter, one expects an increase in antagonism with a decrease in available nutrients. These theoretical contemplations are addressed in this study.

Many bioactive compounds come in the form of enzymes, e.g., colicins and pyocins (22). These proteinaceous, toxic compounds are often produced against closely related strains; e.g., colicins kill Escherichia coli and pyocins kill pseudomonads. Many of these compounds have DNase activity (22), indicating a role for DNases in competition. This is further illustrated in several studies showing that extracellular DNases (exDNases) and effectors of these have roles in hydrolyzing extracellular DNA (exDNA) in situations like biofilm development, toxin production in the presence of exDNA, in enriched plant root-associated bacteria (23–27), and are even differentially selected for by crop plants (28). Therefore, we further hypothesize that exDNase production has an important role in the structure of bacterial interaction networks.

In the study presented here, we compare two populations of antagonists and provide support for the CSIC model. In addition, we also determine the role of exDNase production in coexistence using mathematical modeling approaches.

## RESULTS

### Bacterial load and incidence of antagonism correlate positively.

**FIG 1:**
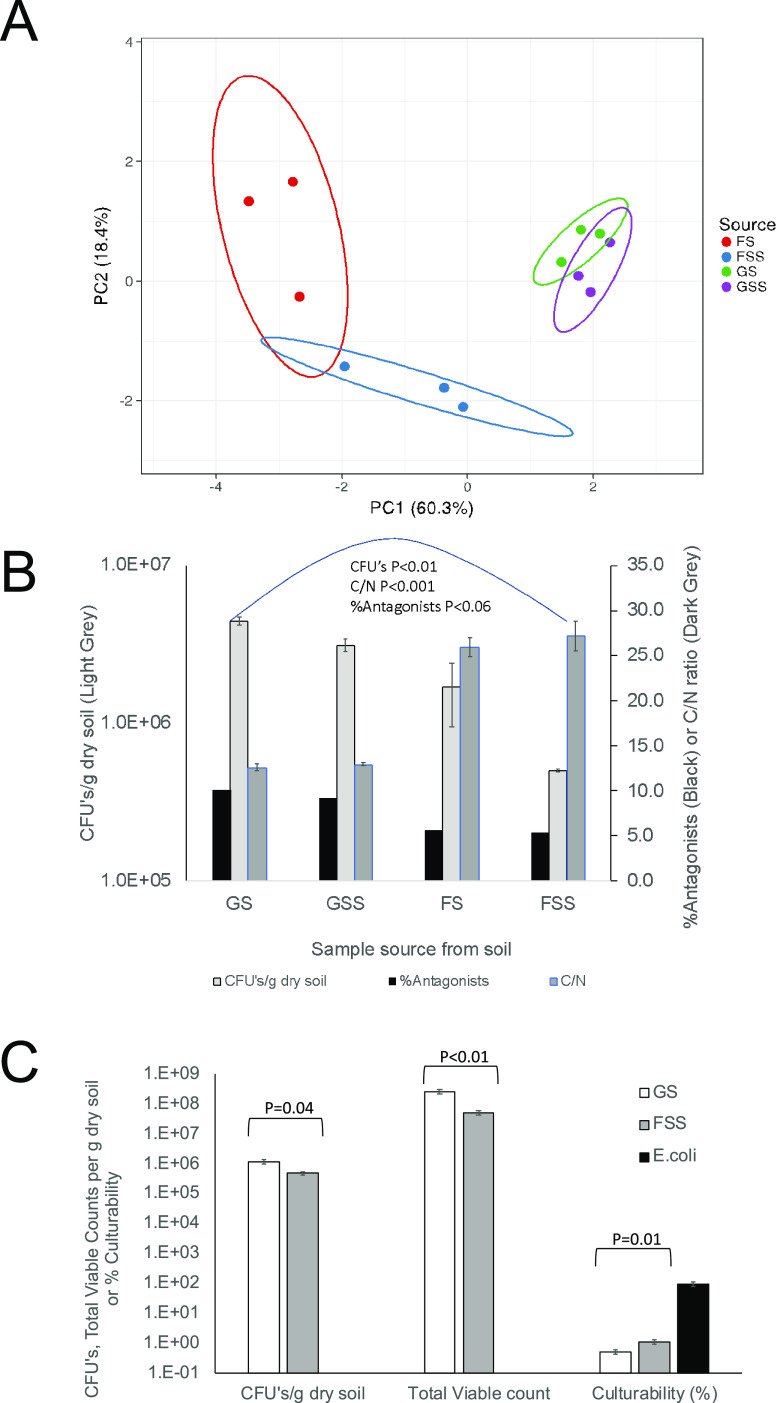
Visualization of the quantification of soil edaphic characteristics. (A) PCA of the soil samples using the edaphic characteristics pH, %N, %C, C/N ratio, temperature, water content, CFU/g dry soil, and % antagonists. Colored ovals represent the 95% confidence interval (CI). (B) Averages of CFU/g dry soil of samples (light gray) and C/N ratio (dark gray). Error bars represent SEM (*n* = 3). Black bars represent the pooled % antagonist values. The data show strict correlation between CFU/g dry soil, pooled % antagonists, and C/N ratio. (C) Estimation of total viable bacterial cells in GS and FSS and the culturability of these populations on 10% TSA. Error bars represent SEM (*n* = 6). In E. coli, the culturability was 95.5% ± 13.5% (*n* = 12).

In order to obtain support for the CSIC or the NDSIC model, it was initially hypothesized that with an increasing bacterial load in an environment, the relative presence of antagonists increases due to closer proximity of competing bacteria, which would benefit antagonists. This would result in an increased presence of these antagonists. In order to probe whether the presence of competitors is a compulsive factor for toxin production, as would be the case in a CSIC model, the effect of the environmental bacterial load on the relative number of antagonists on Staphylococcus CWZ226 or E. coli MC4100 was determined. Soil samples from the Smith College MacLeish Field Station in Whately, MA (29), were retrieved from the grassland surface (GS; 3-cm depth), the grassland subsurface (GSS; 15- to 20-cm depth), the organic (O) horizon from hemlock forest soil (FS; 3-cm depth), and the forest subsurface (FSS; A/B horizon, 15- to 20-cm depth). To elucidate key factors distinguishing soil environments and their impacts on the bacterial load and percentage of antagonists, we determined edaphic characteristics like pH, percent nitrogen (%N), percent carbon (%C), carbon-to-nitrogen ratio (C/N), temperature, water content, CFU/g dry soil, and percentage of antagonists (Table S2-1 in the supplemental material). Principal component analysis (PCA) showed that principal component 1 (PC1) and PC2 explained 78.7% of the variation and that there was no overlap between FSS and GS samples (Fig. 1A). The bacterial load in each environment, expressed in CFU per gram (dry) weight, decreased from (4.41 ± 0.26) × 10^6^ (mean ± standard error of the mean [SEM]) in GS to (3.12 ± 0.29) × 10^6^ in GSS and from (1.68 ± 0.73) × 10^6^ in FS to (5.01 ± 0.10) × 10^5^ in FSS. The bacterial load in GSS was lower than that of soil taken from the surface (*t* test, *P* < 0.02). Similarly, the bacterial load in the hemlock O horizon was higher than that in the A/B horizon (one-sided heteroscedastic *t* test, *P* < 0.12). Furthermore, grassland samples always had a higher bacterial load than forest samples (Table S2-1). Moreover, culturability for GS and FSS was 0.53% ± 0.08% and 1.12% ± 0.19%, respectively (*P* = 0.01, *t* test). The total viability results also indicate that GS had a higher bacterial load than FSS (Fig. 1C). The differences in CFU/g dry soil determined on 10% tryptic soy agar (TSA) plates, though compromised, reflected the *in situ* bacterial loads.

To determine the relative numbers of antagonists, 517, 494, 492, and 462 (*n* = 5 different dilution series per sample site) colonies from GS, GSS, FS, and FSS, respectively, were transferred to indicator lawns containing Staphylococcus or E. coli. Of these colonies, 10.3%, 9.3%, 5.7%, and 5.4% (53, 46, 28, and 25 isolates) inhibited the indicator lawn (*P* < 0.06 for GS versus FSS, *F* test) (Fig. 1B). The results obtained from mock communities showed that the method employed gave the expected ratios and, thus, allowed us to conclude that the level of antagonism reflected the soil community (Text S1).

The difference between GS and FSS was always substantial for pH, %N, %C, and the C/N ratio, with *P* values of <0.002 (heteroscedastic), <0.05, <0.01, and <0.001 respectively (*t* test). However, as expected, only the C/N ratio strongly reflected the sample sites and bacterial loads (Fig. 1B). The C/N ratios were 12.6 and 13.0 in GS and GSS, respectively (*P* = 0.24), whereas the samples from the forest soils had C/N ratios of 25.9 and 27.2 (*P* = 0.26). The C/N ratio is a well-known parameter indicating bacterial load, and the results support the hypothesis that available nitrogen and other quality nutrients allow a higher bacterial load (30–35), leading to increased competition that boosts the population of toxin-producing bacteria. Separating the parameters of competitor presence and nutrition depletion to more clearly understand the cues for bacterial toxin production warranted further study. To do so, we contrasted the GS and FSS populations for their connectance, response to nutrients, and ability to coexist, since these populations represented the two samples with the greatest deviations in terms of CFU/g dry soil, percentage of antagonists, and C/N ratio.

### Support for CSIC.

**FIG 2:**
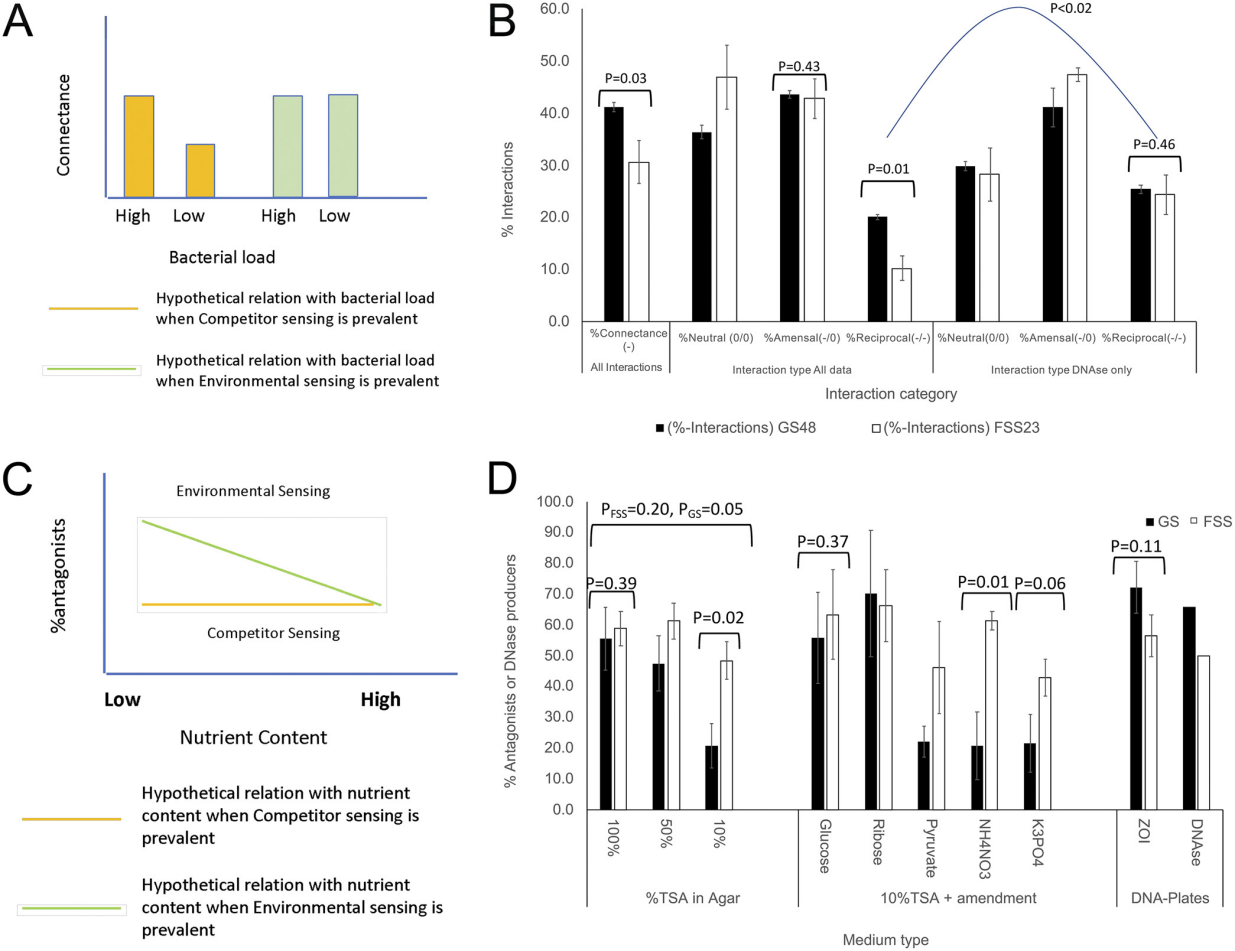
Differentiation of the CSIC and NDSIC models. (A) Theoretical considerations and hypothetical outcome for connectance when competitor sensing (yellow bars) or environmental sensing (green bars) is the major cue for bacteriogenic toxin production. (B) The empirical connectance in both populations with interaction types in the total and exDNase-producing populations (black bars, GS_48_; white bars, FSS_23_). The data indicate a higher connectance in the GS population than in the FSS population, thus giving support for the CSIC model. The rates of neutral (0,0), one way antagonism (0/-), and reciprocal antagonistic interactions (-/-) indicate more reciprocal interactions in the GS population than in the FSS population. This difference is not found in the exDNAse-producing population. (C) Theoretical considerations and hypothetical outcome to a response to a decrease in nutrients when competitor sensing (yellow line) or environmental sensing (green line) is the major cue for toxin production. (D) Responses of the GS and FSS populations to a decrease in nutrient strength and to nutrient complementation, as well as to DNA plates to test for DNA availability and DNase activity. Error bars represent the SEM (*n* = 3).

To further obtain support for the CSIC or the NDSIC model, the presence of a competitor and the role of available nutrients in toxin production by the GS and FSS populations were determined. To do so, we (i) determined the connectance of both populations and (ii) determined if these populations responded differentially to a reduction of available nutrients (Fig. 2). The connectance was 41.2% ± 0.9% for the GS population and 30.6% ± 4.2% for the FSS population (*P* = 0.03) (Fig. 2B). To address the response to nutrients (Fig. 2D), the isolates from both populations were tested on 100%, 50%, and 10% TSA. On 100% TSA, the difference between the FSS and GS populations was negligible (*P* = 0.39). The relative amount of antagonists in the FSS population decreased from 58.9% to 48.4% on 100% and 10% TSA, respectively (*P* < 0.14, sign and *t* test). The relative amount of antagonists in the GS population decreased from 55.6% to 20.8% on 100% and 10% TSA, respectively (*P* < 1 × 10^−8^, sign test, or *P* = 0.02, *t* test). The reduced nutrient content in a complex medium like TSA and the corresponding reduction in antagonist activity may be caused by depletion of a number of nutrients. Therefore, we tested whether the addition of 100 mM glucose, 120 mM ribose, 200 mM pyruvate, 50 mM NH_4_NO_3_, or 100 mM K_3_PO_4_ would complement the lack of C, N, or P in the 10% TSA plates and restore the levels of antagonism. Indeed, the addition of glucose or ribose to 10% TSA increased the antagonism to levels like those on 100% TSA, mainly for the GS population (*P* = 0.05 for GS on glucose). Interestingly, pyruvate did not complement the reduced sugar content in 10% TSA. Inorganic phosphate also failed to increase the levels of antagonism to the levels on 50% or 100% TSA. Ammonium nitrate also did not do so for the GS population (*P* = 0.5), and only an effect for the FSS population was observed (*P* = 0.06). Although there was a correlation with available nutrients, the data indicated that it was the increase of select nutrients that supported antagonist activity, not a decrease as expected in the NDSIC model.

In order to test whether the aggressiveness of a population correlated with its ability to produce a zone of inhibition (ZOI), we determined the aggressiveness index (AI) for all strains according to Zapien-Campos et al. (15) and contrasted the average AI of those strains producing a ZOI to the average AI of those not able to produce a ZOI (Table 1). We expected those populations producing a ZOI to have a higher aggressiveness index than those that did not. Accordingly, populations able to produce a ZOI almost always had a higher AI (0.4 to 8.7) than those strains not producing a ZOI on 100% TSA, 50% TSA, and 10% TSA (−0.8 to −3.5). Even though the number of GS isolates producing a ZOI on 10% TSA was limited (*n* = 6), the AI of this population stood out (8.7) compared to the AIs of the rest (0.4 to 3.4). The main outlier was the FSS population when tested on 10% TSA, for which the population producing a ZOI had a negative AI (−0.3) and the population not producing a ZOI had a positive AI (0.3, *P* = not significant). Only in this population, when tested on 10% TSA, was the expected correlation of ZOI and AI not observed.

**TABLE 1:** Aggressiveness index values of populations of isolates producing a zone of inhibition on different media

Medium or test	Soil source	Value with^*a*^:						*P* value^*b*^
		No ZOI			ZOI			
		AI	SEM	No. of isolates	AI	SEM	No. of isolates	
% TSA								
100	GS	−0.8	3.6	19	0.4	2.0	29	NS
	FSS	−2.3	3.4	8	1.2	1.4	15	0.2 > *P* > 0.1
50	GS	−3.5	2.0	24	3.4	3.0	24	*P* < 0.05
	FSS	−2.6	3.9	7	1.5	1.4	15	0.2 > *P* > 0.1
10	GS	−1.3	1.9	42	8.7	6.7	6	*P* < 0.05
	FSS	0.3	1.9	12	−0.3	2.4	11	NS
10% TSA plus:								
Glucose	GS	−1.2	2.4	20	0.7	2.7	28	NS
	FSS	−3.4	2.7	8	1.8	1.6	15	*P* < 0.05
NH_4_NO_3_	GS	0.4	2.1	42	−4	2.3	5	NS
	FSS	−4.7	1.8	9	3	1.7	14	*P* < 0.01
K_3_PO_4_	GS	0.7	1.9	43	−3.6	9.6	4	NS
	FSS	−3.2	1.9	12	3.5	1.9	11	*P* < 0.02
DNA plate^*c*^								
Test for killing	GS	−3.1	4.2	10	0.7	2.1	38	NS
	FSS	1.4	2.3	11	−1.3	1.9	12	0.2 > *P* > 0.1
Test for DNase activity	GS	−5	4.1	10	1.2	2.1	38	0.1 > *P* > 0.05
	FSS	1.3	2.6	10	−1	1.8	13	NS
								
Test for killing in DNase producing populations	GS	−19	1	2	1.1	2	36	*P* < 0.02
	FSS	10	NA	1	−0.8	1.1	12	

a ZOI, zone of inhibition; AI, aggressiveness index.

b Return is from the homoscedastic, one-sided *t* test. NS, not significant with *P* > 0.2.

c Using DNA-plates seeded with *Staphylococcus* CWZ226 allows to test for a ZOI and the ability to hydrolyze DNA in the whole populations, as well as the exDNase producing populations.

The addition of glucose to 10% TSA restored the AIs to the levels found for the populations producing a ZOI for the GS and FSS populations, with the AIs found to be like those of populations tested on 50% and 100% TSA. In contrast, when NH_4_NO_3_ was added to 10% TSA, the AI of the FSS population was very much restored, and the difference in AIs of populations able and unable to produce a ZOI increased even more than found on 100% TSA (*P* < 0.01). However, NH_4_NO_3_ strongly negatively affected the AI of the GS population, and the expected correlation between AI and the ability to produce a ZOI was not observed. Most interestingly, the GS populations producing a ZOI on 10% TSA and 10% TSA plus NH_4_NO_3_ must have been different populations even though the levels of antagonism were similar in both populations. Indeed, the two populations had only two isolates in common (Tables S3-1 to S3-3). Therefore, we concluded that although the addition of sugar to 10% TSA restored the AI for both populations, it was nitrogen availability under low-nutrient conditions that regulated the production of toxins in subpopulations derived from the GS soil.

While exDNA may act as an important source of nutrients (27), it also indicates the presence of competing bacteria (28). Given this association, we hypothesized that if competitor sensing was the primary instigator for toxin production (over nutrient availability) in the GS and FSS populations, then antagonism would increase when these populations were plated on DNA plates (Difco) compared to the antagonism on 100% TSA. We found substantial support for the idea that exDNase production was important in antagonism. First, a strong correlation between the ability to produce a ZOI on Staphylococcus and exDNase activity was observed in both GS and FSS populations (*P* = 9.84 × 10^−12^ for GS and *P* = 7.75 × 10^−7^ for FSS). Almost all isolates that antagonized on DNA plates were also exDNase producers (Tables S3-1 to S3-3). Second, substantially more GS isolates antagonized Staphylococcus on DNA plates than on TSA (76.5% versus 62.3% respectively), which was reversed for the FSS population (52.0% on DNA plates and 64.0% on 100% TSA) (Fig. 2D), indicating the larger role for exDNases in a high-bacterial-load and low-C/N-ratio environment than in a low-bacterial-load and high-C/N-ratio environment.

The AI levels obtained for the GS populations producing a ZOI or hydrolyzing exDNA on DNA plates were higher than those of the populations not producing a ZOI (*P* = not significant) or hydrolyzing DNA, exactly as expected (*P* < 0.1). However, for the FSS population, this was reversed. Even when the DNase-producing populations only (GS_38_ [the 38 exDNase-producing isolates in the GS population] and FSS_13_) were analyzed, these relationships did not change.

### Reciprocal amensal interactions are underrepresented in the FSS population.

In addition to the high connectance in the GS population, the total amount of reciprocal (two-way amensal) interactions was higher in the GS population (20.1% ± 0.5%) than in the FSS population (10.3% ± 0.5%, *P* = 0.01) (Fig. 2B), while the level of amensal interactions was very similar in both populations (42.8% to 43.6%, *P* = 0.43) (Fig. 2B). However, in the GS population, the percentage of reciprocal interactions, estimated by taking the square of the amensal interactions (0.436^2^ = 19.0% ± 0.7%), was almost equal to the number of observed interactions (20.1% ± 0.5%, *P* = 0.13). Interestingly, in the FSS population, the estimated percentage of reciprocal interactions (0.428^2^ = 18.3% ± 3.1%) was higher than the observed percentage of reciprocal interactions (10.3% ± 2.4%, *P* = 0.05). Therefore, the reciprocal amensal interactions were underrepresented in the FSS population. In addition, exDNase activity was increased in the isolates involved in reciprocal interactions, which for the FSS population were 10.3% ± 2.4% and 24.4% ± 3.8% (*P* < 0.02) of the total and DNase-producing populations, respectively. For the GS population, the amounts were 20.1% ± 0.5% and 25.4% ± 0.8% of the total and exDNase-producing population, respectively (*P* < 0.01) (Fig. 2D). The relative amounts of exDNase-producing isolates involved in reciprocal antagonism were very similar (Fig. 2B) (*P* < 0.46).

### Coexistence is differentially affected by DNase production.

**FIG 3:**
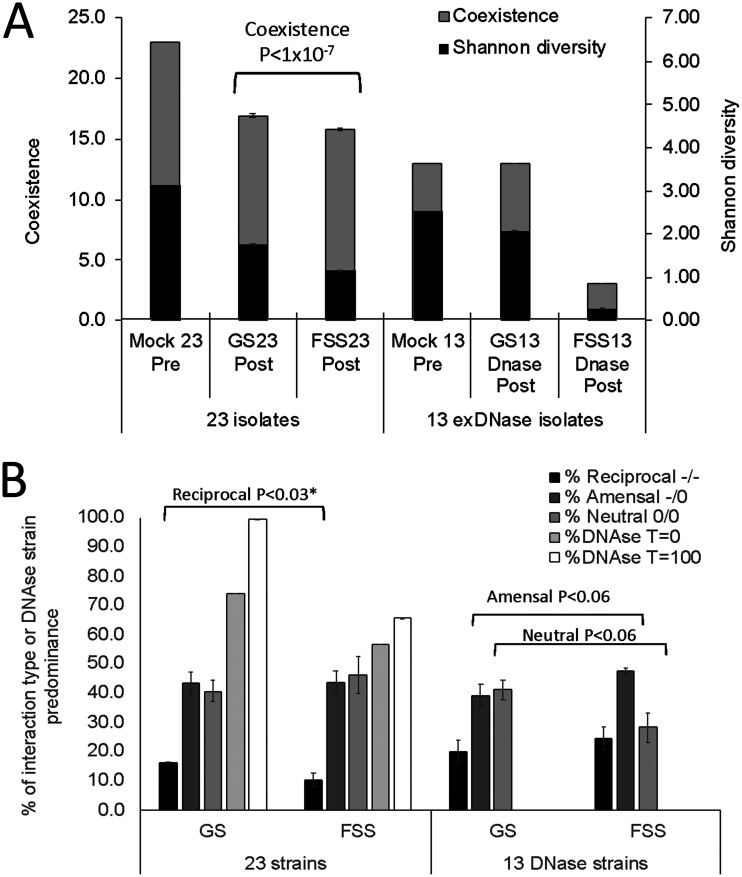
Diversity and coexistence in GS and FSS populations pre- and postsimulation. (A) Estimated diversity and coexistence pre- and postsimulation for the whole (23 isolates) and the DNase-producing (13 isolates) population using a probabilistic cellular automaton (ProbCA) and the interaction matrices (Tables S3-1 to S3-3). The forest subsurface (FSS_23_) population was composed of 23 isolates, and the grassland surface (GS_23_) population was composed of the 23 best-surviving isolates selected after a pilot run with the full data set. Coexistence is the number of isolates present in the dynamic equilibrium. Shannon diversity was based on the resulting isolate distribution after simulation. Error bars represent the SEM (*n* = 100). SEMs in the GS_13_ and FSS_13_ populations were too small to show. (B) Interaction types and relative abundances of exDNase-producing isolates pre- and postsimulation in the 23-strain and 13-exDNase-producing-strain-only matrices. *, heteroscedastic *t* test; all others were homoscedastic.

Competitive (12, 14) and (a)social interactions in combination with media (5) positively affect coexistence. Additionally, killing is a means to promote diversity (16), and connectance as well as reciprocal interactions were higher in the GS than in the FSS population. Therefore, we predicted coexistence in the GS population to be higher than in the FSS population. In order to test for coexistence, we employed an *in domum*-developed probabilistic cellular automaton (ProbCA) (Text S4). Due to differences in starting sizes, the GS_48_ population was simulated twice in series, as follows: (i) the population as a whole (48 × 48 comparisons) and (ii) GS_23_, consisting of the 23 best-surviving isolates, representing 99.2% of all cells occupied at the end of the first simulation. As shown by the data in Fig. 3A, a 350 × 350 matrix seeded with GS_23_ or FSS_23_ led to a decrease in coexistence in both populations in which 16.9 ± 0.2 and 15.8 ± 0.1 isolates coexisted, respectively, although the difference in coexistence was only 1.1 isolates (7%, heteroscedastic one-sided *t* test, *n* = 100, *P* < 1 × 10^−7^). Shannon diversity decreased in both populations relative to that in the not-simulated mock population of 23 at time zero (*T* = 0) and was lower in the FSS_23_ population than in the GS_23_ population (1.16 and 1.77 respectively, *P* < 1 × 10^−136^). Because the contributions of exDNase populations from GS and FSS to reciprocal interactions were essentially the same (*P* = 0.46) (Fig. 2B), we expected simulations of the exDNase-producing populations from both samples to lead to similar levels of coexistence. Indeed, the occupancy (number of cells occupied by exDNase-producing isolates divided by the total number [350 × 350 = 122,500]) of exDNase isolates increased in both populations after the simulations. In the GS_23_ population, this increased from 73.9% to 99.5% (a 34.6% increase), and in the FSS_23_ population, it increased from 56.5% to 65.6% (a 16.1% increase) (Fig. 3B). Clearly, producing exDNases was an advantage; however, the advantage was greater in the GS population.

To examine the consequences of antagonism by exDNase-producing isolates only, simulations were run using the 13 exDNase isolates from the FSS population (FSS_13_) and the best-surviving 13 isolates from the GS population (GS_13_), representing 99.6% of the 38 exDNase-producing isolates in the GS population (GS_38_; FSS_13_ is 100%) after a pilot run. Compared to the results of the GS_23_ and FSS_23_ simulations, three major differences can be observed (Fig. 3A). (i) The coexistence of the GS_13_ population was unchanged compared to the start situation and remained 13 (Fig. 3A, gray bars). (ii) The Shannon diversity of GS_23_ (1.8 ± 0.002) increased in GS_13_ despite starting with fewer isolates and was 2.08 ± 0.0004 (*P* < 1 × 10^−97^). Therefore, the GS_23_ diversity was positively affected by the exDNase-producing population. In contrast, (iii) the Shannon diversity in FSS_23_ (1.16 ± 0.001) was higher than that in FSS_13_ (0.28 ± 0.003) (*P* < 1 × 10^−123^), while the coexistence was low (three strains). Two opposite effects were observed in both populations: in the GS population, exDNase production supported diversity and coexistence, while in the FSS population, it did not.

The low coexistence in the dynamic equilibrium in the FSS_13_ simulations, not seen in the FSS_23_ (15.8 strains), GS_23_ (16.9 strains), and GS_13_ (13 strains) simulations (Fig. 3B), was associated with a low rate of neutral interactions and more amensal and reciprocal interactions in the FSS_13_ population only (Fig. 3B). In contrast, the higher rate of neutral interactions and lower rates of amensal and reciprocal interactions in the GS_23_, FSS_23_, and GS_13_ populations led to higher levels of coexistence. Using the Dixon test, we identified the reciprocal interactions in the FSS_13_ populations as lower than in any of the other sample origins (*P* > 0.30), as well as the rate of 28.2% of neutral interactions (0.05 > *P* < 0.10). For the amensal interactions, the outlier was GS_13_ (0.2 > *P* < 0.1).

### Phylogenetic distance negatively affects the rate of amensalism.

**FIG 4:**
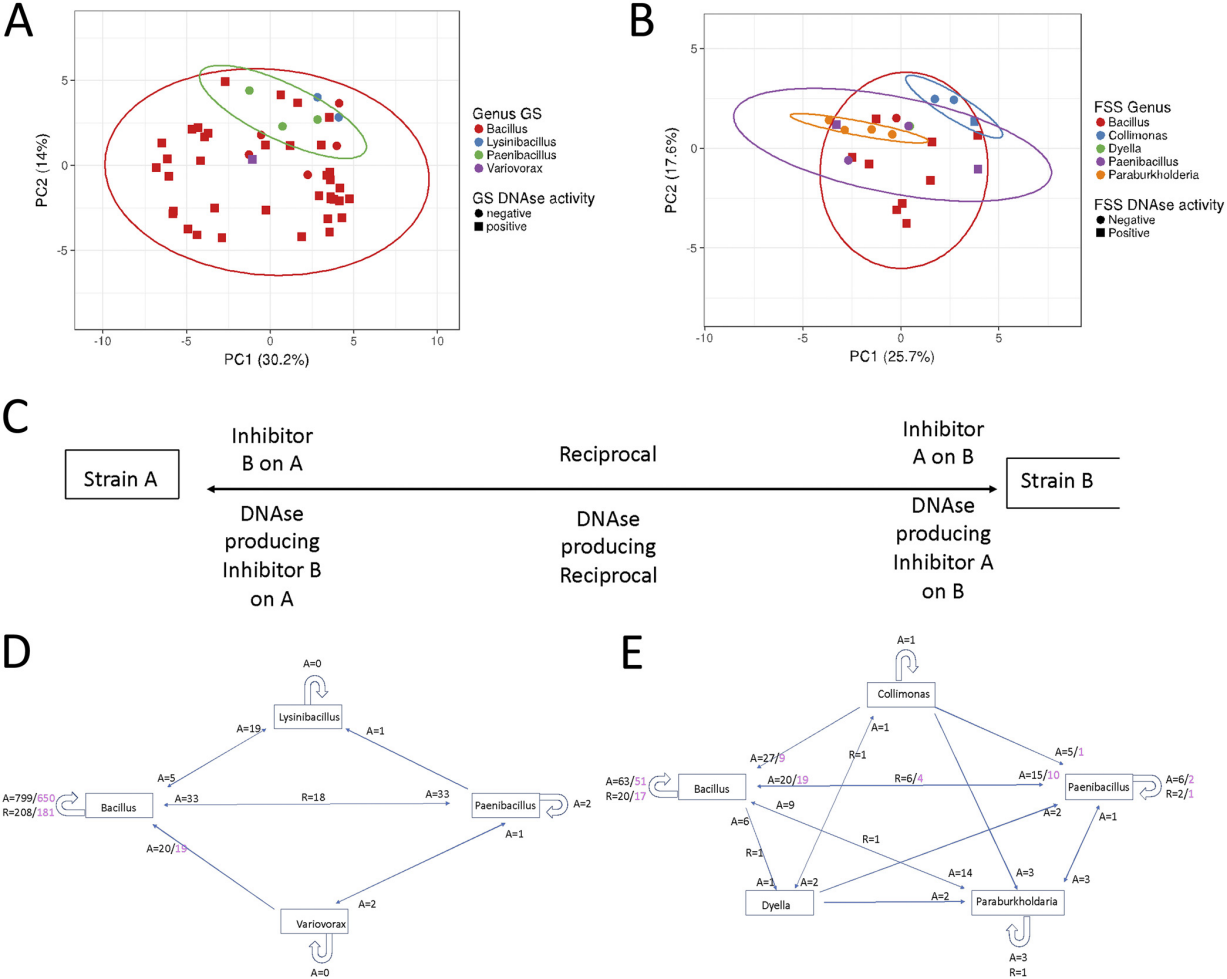
Visualization of interactions in the GS_48_ and FSS_23_ populations using PCA (A and B) and schematic models (C, D, and E). The GS_48_ and FSS_23_ majority rule consensus interaction matrices were used as input for the PCA using default settings. (A) First and second component of the PCA analysis of the GS_48_ interaction matrix. All variation falls within the 95% CI of Bacillus (red line). (B) First and second components of the PCA analysis of the FSS_23_ interaction matrix. All variation falls within the 95% CI of Bacillus (red line). (C) Explanatory diagram of the meaning of the interactions between different genera in panels D and E. (D) Interaction diagram of the GS_48_ population. (E) Interaction diagram of the FSS_23_ population. A, amensal interactions; R, reciprocal interactions. In black are all interactions, and in purple are all exDNase-associated interactions.

To model patterns of amensalism and reciprocal amensalism, we first determined the genera of the isolates using 16S rRNA sequencing followed by BLAST searches of the complete-genome databases at NCBI. The results (Table S5-1) showed that the GS population consisted of 1 Gram-negative isolate and 47 Gram-positive isolates in four genera. The FSS population consisted of 8 Gram-negative isolates and 15 Gram-positive isolates in five genera. Based on genus, GS was less diverse than FSS, having 48 isolates from four genera while FSS contained 23 isolates from five genera (Fig. 4). In both populations, Bacillus was predominant, comprising 42 (87.5%) and 11 (47.8%) isolates in the GS and FSS populations, respectively. The second predominant genus was Paenibacillus, with three and four isolates from GS and FSS, respectively. The GS population contained two Lysinibacillus isolates and a Variovorax isolate, and the FSS population contained four Paraburkholderia, three Collimonas, and one Dyella isolate.

To compare the variations of patterns of inhibition of the indicator strains, PCA plots were created using the interaction matrices. As shown by the data in Fig. 4A and B, PC1 and PC2 explained 44.2% and 43.3% of the variation for GS and FSS, respectively. The vast majority of isolates, regardless of genus, fell within the 95% confidence interval (CI) of Bacillus in both populations. We found 90% of all Bacillus isolates in both populations to produce exDNases. Bacillus thuringiensis is known to produce toxins in the presence of DNA and is a species in the Bacillus cereus group known for toxin producers to which many of our isolates are related (Table S5-1). Furthermore, many Bacillus species produce lipopeptides, compounds with antimicrobial activity involved in biofilm restructuring (36). When including the third component, >52% of the variation could be explained in both populations, and the same conclusions were supported (Fig. S6).

The interaction diagrams indicating inter- and intrageneric interactions, as well as those that were exDNase mediated, showed that the majority of intrageneric interactions was within the genus Bacillus in both populations (Fig. 4C to E) but was much more profound in the GS than in the FSS population. The number of interactions between Gram-positive producers on Gram-positive indicators was overrepresented. Gram-positive producers and Gram-negative indicators, as well as Gram-negative producers and indicators, were underrepresented in both populations (χ^2^, df = 3, *P* < 0.001).

In both populations, the observed amensal and reciprocal interactions were different from the expected (χ^2^, df = 3, *P* < 0.001). Most intergeneric interactions took place between Bacillus and Paenibacillus in both populations. Furthermore, intrageneric reciprocal interactions were overrepresented in both populations (χ^2^, df = 3, *P* < 0.01). This was especially clear in the FSS population, where the reciprocal interactions between the Bacillus isolates (36.4%), as well as the Paenibacillus (33.3%) and the Paraburkholderia (16.7%) isolates, were overrepresented relative to the intergeneric interactions (4.9%) and total number of reciprocal interactions (12.6%). Surprisingly, in the GS population, all intrageneric reciprocal interactions were found in the Bacillus genus alone (24.2%). The intergeneric interactions were 9.4%, and total interactions were 20.0%.

Modeling did not affect the genera present even though the number of isolates present in the dynamic equilibrium decreased compared to the starting condition.

## DISCUSSION

At the outset of this work, our theoretical contemplations based on theoretical soil microbial and diversity models, such as presented by Curtis and Sloan (20), and the competition models reviewed by Hibbing (21), were considered insufficient; however, they provided a framework for hypothesis development and testing. After the initiation of this project, we found a strong correlation between the CFU/g dry soil and the percentage of antagonists. Although this correlation supports a CSIC over an NDSIC model for the production of toxins, a correlation with the C/N ratio was also found. Though the C/N ratios strongly support the relative order of CFU/g dry soil, the results lay bare a conundrum: is the main cue to toxin production the nutrient status of the soil or the presence of competitors? Resolution of this conundrum is provided by (i) the increase in connectance in the population with a higher bacterial load (GS), (ii) the reduction of antagonist activity with decreasing nutrients, (iii) the higher rate of reciprocal amensal interactions in the population derived from a high bacterial load, and (iv) the frequency with which pathways for the production of secondary metabolites become cryptic outside the context of their natural environment—e.g., storage in glycerol or growth on agar plates (attenuation) lowers toxin expression (37–39), but toxin expression is reestablished when the correct conditions are met, including the presence of a competitor. Here, for example, of the 78 (53 + 25) isolates tested, 97.4% produced a ZOI on indicator isolates within the same population (Tables S3-1 to S3-3), which was an increase from the 44.0% to 64.4% on Staphylococcus only. (v) Finally, isolates producing a ZOI tended to have a higher aggressiveness index (AI) than the strains not producing a ZOI, especially for the GS population on TSA and DNA plates.

However, the complementation studies showed that nutrients did play a role at lower nutrient levels in the media. Glucose and ribose restored the levels of antagonism for mainly the GS population, while NH_4_NO_3_ did this for the FSS population. Interestingly, NH_4_NO_3_ addition reversed the AI in the GS population, with a higher AI for those strains not producing a ZOI than for those producing a ZOI. This may be explained by the C/N status of the soils the isolates were derived from. Because of the low C/N status of the GS soil, the addition of different nitrogen sources selectively leads to an antagonist population with low AI when available nutrients are scarce. This probably is a condition under which an antagonist with low AI can get the opportunity to proliferate in a competitive environment. This contrasts with the FSS population. Being isolated from a soil poor in available nitrogen already, nitrogen availability establishes the correlation of AI and the ability to produce a ZOI when available nutrients are limited. This is the condition under which antagonists with high AIs can take the advantage in an otherwise less competitive environment. We have not been able to find support for these models in the literature. In addition, although we obtained an expected order of bacterial loads, which tended to correlate well with the active microbial biomass and enzyme activity (40), CFU/g dry soil on 10% TSA may not be an accurate reflection of the total *in situ* bacterial load. And yet, our data do support that the CFU/g dry soil reflected the total viable bacterial populations in GS and FSS, although with low culturability. Furthermore, while the rates of amensal interactions were the same in the GS and FSS populations (Fig. 3A), more reciprocal interactions were observed in the GS population than in the FSS population, which can be explained as a consequence of random amensal interactions. In contrast, reciprocal amensalism in the FSS population is underestimated and may be selected against. Since participation in reciprocal antagonism is a consequence of the rate of amensalism and randomness, reciprocal antagonism is a more appropriate term than competition, which infers a purpose. This is counterintuitive to the hypothesis that the production of toxins is selected for (e.g., see reference 13). This discrepancy can be explained by the experimental design. De Vos et al. (14) and Kehe et al. (7) did their testing in nonstructured environments as opposed to testing within structured environments (8, 9; this study). In structured environments, the concentrations of toxin are distance dependent, explaining the phenomena we observed. When we assume the distribution between bacteria in soil is homogeneous and only 0.53% and 1.13% of the total soil microbial populations form colonies on 10% TSA, then the average distance between bacterial cells is 12.1 and 32.1 μm for GS and FSS, respectively (Table S7-1). This is well within the range Raynaud and Nunan (41) estimated (0.3 to 532.4 μm) using simulations of thin sections of soil. If the percentages of antagonists of the colony-forming populations are also an accurate estimate in the nonculturable populations, then the average distances between antagonists are 25.8 and 85.0 μm for GS and FSS, respectively. When estimating the volume of a sphere using these distances as the radius, the volume in the FSS sphere is 35.8-fold larger than that of the GS sphere. If these bacteria produce the same amounts of toxin, and the toxin is evenly distributed in this sphere, then the toxin concentration in the FSS sphere is 2.8% (1/35.8) of that in the GS sphere. When the estimated connectance is also corrected for, the volume in the FSS sphere is 48.2-fold larger than that of the GS sphere and the toxin concentration only 2.1% of that in the GS sphere. Compellingly, to be an effective toxin producer requires substantially more effort for the FSS population. Furthermore, we observed that the number of interactions was increased intra- rather than intergenerically, which would further dilute the target in the populations that are more diverse, reducing effective toxin production even further.

Our observation that populations were reduced in antagonist activity under decreasing nutrient content (Fig. 2D) supports the observation by Russel et al. (11) of the trade-off between exploitation and interference competition. However, most isolates were antagonistic at high nutrient content with a competitor present. Therefore, antagonists may be specialists under different conditions and the trade-off in cost is environment dependent (grassland versus forest soils) rather than primarily lifestyle dependent (metabolic, physiologic, or phylogenetic). This is exemplified by our complementation and AI studies, which also show differential responses to C, N, and P availability depending on the source of the population. Although the FSS environment may contain more specialists and exploiters and the GS population more generalists and antagonists, on the level of populations of antagonists, this discussion is futile: All isolates are antagonists. The observation that both populations showed a decrease in antagonism with decreasing nutrient content but that there was a more profound decrease in the GS population than in the FSS population (*P* = 0.02) suggests that antagonists in the FSS population are able to produce toxins at low nutrient availability better than those populations from an environment of high nutrient availability. This again illustrates that the trade-off cost between exploitation and antagonism is not one-size-fits-all.

Our data also indicate a role for exDNase production in the soil environment. The production of exDNases correlated with the ability to inhibit Staphylococcus and was higher in the GS population than in the FSS population. Furthermore, exDNase producers had higher AIs and were competitive in simulations using the GS population, which was not seen in the FSS population. This was also exemplified when populations were tested for antagonism and AI on DNA plates. The strong correlation between toxin production and exDNase activity suggests a functional linkage between, on one hand, increasing the pool of exDNA in the environment and, on the other hand, the utilization of available exDNA. That inorganic phosphorus did not affect the levels of antagonism in both populations supports the idea that organic phosphorus may be the main target of toxin production. In the GS population, this linkage was associated with competition and coexistence. Since our exDNase assay only determined the observable hydrolysis of exDNA, exDNA in soil is considered the target for these exDNases. Various sources of exDNA have been identified, e.g., the sloughing of plant cells from the root tip or, alternatively, the consequence of lysis of bacteria (42), therefore providing the functional linkage. At an increased bacterial load, more isolates may produce compounds with antimicrobial activity by lysing bacterial cells. Lipopeptides produced by Streptomyces, Pseudomonas, and Bacillus (36) kill by forming pores in membranes, lysing competing bacteria, which in turn provide the substrate for the exDNases. In Bacillus, lipopeptides are associated with biofilm restructuring and cannibalism (43). Disruption of biofilms allows better access for antimicrobials to otherwise recalcitrant cells. Variation in geographic lipopeptide production by Bacillus was observed previously (44), and they have a role in competition (36).

Although naturally competent, Bacillus is a genus not particularly known for the production of exDNases (24). However, their importance is illustrated by the following. With a decreasing C/N ratio, it is increasingly likely that phosphorus is limiting, resulting in competition for available organic phosphorus. Similar observations were made by Mulcahy et al. (27), Turk et al. (45), and Kamino and Gulden (28), who isolated exDNase-producing Bacillus strains mainly from soils of low C/N ratios and low phosphorus. Therefore, we hypothesize that a low C/N ratio results in generalist, exDNase-producing Bacillus isolates with high aggressiveness scavenging for organic phosphorus. A high C/N ratio results in fewer of these isolates. Consequently, more and fewer exDNase-mediated reciprocal amensal interactions between closely related bacteria were observed, respectively (7, 11, 14).

Although these explanations are plausible, genetic linkage or coregulation of the expression of toxin and exDNase and the consequences for competitiveness and coexistence are yet to be established. Studies with strains isogenic for exDNase activity have shown a role for exDNases in fitness and virulence (25–27).

On the genus level, diversity was higher in the FSS population than in the GS population pre- as well as postsimulation. However, since competitive (12, 14) and social (5) interactions positively affect diversity and both connectance and reciprocal amensal interactions were higher in the GS than in the FSS population, on the isolate level, we predicted coexistence in the GS population to be higher than in the FSS population. This is exactly what we found, but with only a marginal difference in coexistence (1.1 isolate). We found that coexistence increased in populations with more reciprocal (GS_23_ > FSS_23_) (Fig. 4A) and fewer neutral interactions. In support of this, compared to the not-simulated mock community (Mock_13_), in the GS_13_ simulations, the level of coexistence remained at 13 isolates. This indicates that the population of exDNase-producing antagonists coexists well, which also corresponds to a slight increase in reciprocal interactions compared to the level in GS_23_.

In contrast, the large decrease in coexistence in the FSS_13_ simulations, not seen in the FSS_23_, GS_23_, and GS_13_ simulations, could potentially be caused by an increase in taxonomically different isolates, as proposed by de Vos et al. (14) and Kehe et al. (7). Alternatively, a change from neutral interactions in the FSS_23_ population (from ~47% in FSS_23_ to ~30% in FSS_13_) (Fig. 3B) to reciprocal interactions in FSS_13_ (from ~10% in FSS_23_ to ~27% in FSS_13_) (Fig. 3B), not observed in the GS_23_ and GS_13_ populations, may explain the decrease in coexistence.

The first phenomenon is unlikely since the main variation in antagonist activity in both populations was largely represented by Bacillus only, and thus, the populations were not taxonomically different on the genus level. In the second phenomenon, the shift to higher reciprocal interactions and fewer neutral interactions was relatively small, and therefore, the high number of amensal interactions in the GS_23_, FSS_23_, and GS_13_ populations led to a high coexistence, while in the FSS_13_ population, it was the high rate of reciprocal and low rate of neutral interactions that led to low coexistence. This again does not support findings that competition is important for coexistence in an environment as structured as soil (5), but it supports the work by Mougi (10), whose *in silico* work showed that asymmetry in interactions supports stability. According to these models, the FSS_23_ population is expected to be relatively stable because of fewer reciprocal interactions in this population. In contrast, in the exDNase-only populations, the increase in reciprocal amensal interactions in the FSS_13_ population relative to the levels in the FSS_23_ and GS_13_ populations would lead to low coexistence. Both these consequences were observed. Therefore, we conclude that observable exDNase-producing isolates involved in amensal interactions stabilize a community, leading to an increase in coexistence in competitor-sensing interference competition in a structured environment.

## MATERIALS AND METHODS

### Strains, soil isolates, and culture conditions.

All strains were stored in tryptic soy broth (TSB) (catalog number 211825; Difco) with 20% glycerol at −80°C. Staphylococcus sp. strain CWZ226 (46), Escherichia coli strain MC4100 (47), and Serratia sp. strain CWZ222 (Fig. S8-1) were provided by Dr. C. White-Ziegler (Smith College) and maintained on 100% tryptic soy agar (TSA) (catalog number DF0369-17-6; Difco). Lysobacter antibioticus strain CVAP#2 (L. antibioticus strain ATCC 29479 [48]; provided by Dr. J. Handelsman) and Pseudomonas sp. strain CVAP#3 (46) and all isolates from the soil were maintained on 10% TSA (from 100% TSA amended with agar [catalog number DF0812-17-9; Difco]; the final agar concentration was 1.5%). All strains were incubated for 36 to 48 h at 25°C and kept at 4°C until use for a maximum of 1 week.

### Field sites and sampling.

Samples were taken from the Smith College MacLeish Field Station in Whately, MA (29), on 21, 22, and 24 September in 2015. The geographic coordinates for the grassland soil samples are N42°26.983′, W072°40.820′. The hemlock forest soil samples were taken at coordinates N42°27.328′, W072°40.926′ (Garmin eTrex 20x). Soil and air temperatures were taken at the moment of sampling. Soil samples were taken using a sterile spatula or spoon while wearing alcohol-sterilized gloves. The samples were stored in sterile wide-mouth Mason jars (450 mL) and frozen at −20°C upon arrival in the laboratory after subsamples were taken for bacterial counts.

### Determination of CFU/g dry soil and identification of isolates that produced bioactive compounds.

Within 6 h of taking the sample, 1 g of soil was weighed and a suspension was made in 9 mL of sterile phosphate-buffered saline (PBS) (product number 2810305; MP Biomedicals) in a sterile 15-mL conical tube (7). Amounts of 100 μL of a 10-fold dilution series were spread on 10% TSA (6, 29) and incubated for 36 to 48 h at 25°C, after which CFU were counted and plates were stored at 4°C till further use. All plates, with an average number per dilution of 30 ≦ CFU ≦ 300, were used to determine the bacterial load (49). The bacterial counts were corrected for water content, and the bacterial load in CFU/g dry soil was calculated for every plate. Subsamples, dilution series, and plate counts originating from the same soil sample were pooled. The resulting average values per sample site were used to calculate a grand average for an estimation of CFU/g dry soil for a sample period.

To identify the level of antagonism in a soil, colonies originating from the dilution plates used for counting were transferred to master plates made of 10% TSA. Colonies were picked randomly with a sterile flat toothpick. Only plates with fewer than 300 colonies were sampled. Up to 125 colonies from a dilution series representing one sample were transferred to the master plate (10% TSA) and incubated at 25°C for 36 to 48 h. Indicator plates were seeded as follows: a colony was resuspended in 1.0 mL PBS with a sterile synthetic-tipped applicator (catalog number 23-400-122; Fisherbrand), spread over the surface of an agar plate, and dried. Drying is required to prevent swarming of colonies and contamination of neighboring colonies. After drying, Staphylococcus and E. coli were transferred onto the indicator plates as negative controls. L. antibioticus and Pseudomonas sp. strain CVAP#3, which inhibit Staphylococcus, were used as positive controls. Plates were stored at 4°C for 3 to 5 days and incubated at 25°C overnight. Isolates inhibiting an indicator lawn were purified at least twice, retested, stored at −80°C in TSB with 20% glycerol, and given an identifying number (Chris Vriezen antibiotic producer number [CVAP#]). The relative number of antagonists was determined by dividing the final number of isolates producing a ZOI by the original number transferred and tested. All data from the same sample period and site were pooled (7, 12, 14).

### Estimation of the total viable cells and culturability.

Culturability is the fraction of CFU relative to the total population of viable bacterial cells in a sample (50). In order to estimate the culturability in the samples derived from the grassland surface, the forest subsurface, and E. coli suspensions in PBS, we determined the number of viable cells using live/dead stain (BacLight bacterial viability kit, product number L7012; Molecular Probes). On 26 and 28 August 2022, soil samples were taken and dry weight determined as described above. Soil suspensions (1:10) were made in 1× PBS. The suspensions were vortexed for 30 s, and CFU/g dry soil determined. In addition, 100 μL resuspended soil was mixed 1:1 in PBS containing 3 μL Syto9 and 3 μL propidium iodide per mL. Using a Neubauer counting chamber, the number of bright green cells was counted at ×200 or ×400 total magnification on a Leica DM5500B using the I3 filter cube. Only bright fluorescent green cells were counted. At least three different subsamples were counted for every suspension. At least three frames per sample were completely counted, with a minimum of 100 cells per prep. This resulted in six estimates per soil. Control suspensions of E. coli strain MC4100 growing on 10% TSA in PBS were treated in a similar manner.

### Determination of basic soil edaphic properties.

To determine the water content, three soil subsamples for each original sample were weighed, stored at 55°C for 2 to 4 days, and weighed again. The water content is the weight lost after drying divided by the original weight and expressed as a percentage. Dried samples were sieved (product number 04-881G; Fisher Scientific), and the percent nitrogen (%N), percent carbon (%C), and C/N ratio determined using a vario micro select CHNOS element analyzer (51). These measurements were carried out at the Center for Aqueous Biogeochemistry Research at Smith College. For pH determination, dried soil was diluted 1:9 (wt/wt) in demineralized water. After 2 h, the pH was measured using an Accumet model 10 with Accumet probe 13-620-285.

### Nutrient and DNase activity determination.

The following media were used to test the response of the soil isolates: 100% TSA (catalog number DF0369-17-6; Difco) and 50% and 10% TSA (from 100% TSA amended with agar [catalog number DF0812-17-9; Difco], final agar concentration of 1.5%). Nutrient complementation studies were performed by the addition of 100 mM glucose, 120 mM ribose, 200 mM pyruvate, 50 mM NH_4_NO_3_, or 100 mM K_3_PO_4_ to 10% TSA, and the pH was set at 7.3 using HCl or NaOH as needed.

To test for antagonist activity toward Staphylococcus and for exDNase activity, DNA plates (catalog number 263220; Difco) were seeded with Staphylococcus as described above, and soil isolates were tested for their ability to inhibit Staphylococcus and to break down DNA in the agar plates. The positive control for exDNase activity was Serratia sp. CWZ222, or Pseudomonas sp. strain CVAP#3 for a ZOI on Staphylococcus (Fig. S8-1), and the negative-control Staphylococcus colonies were transferred and spotted on the plate using a sterile flat toothpick. Plates were incubated overnight at 25°C, and the zone of inhibition (ZOI) was determined, as well as the exDNase activity by flooding the plate with 1 N HCl (23).

### Determination of connectance and AI.

To determine the connectance (10) of a population, a colony used as the indicator was used to seed a 100% TSA plate as described above. Soil isolates were transferred onto this indicator strain along with controls, and plates were stored at 4°C for 3 to 5 days. After overnight incubation at 25°C, the plates were analyzed for the appearance of a ZOI. Data were recorded as positive (+) when a ZOI was produced or negative (−) when no ZOI was produced. This procedure was done with the GS_53_ population (53 × 53) and the FSS_25_ population (25 × 25). The experiments were repeated three times and quality control (QC) performed, leading to the inclusion of 48 isolates in the GS_48_ population and 23 isolates in the FSS_23_ population (Table 2). The connectance was determined by the number of positive results (an isolate producing a ZOI on an indicator) divided by the total tests performed.

**TABLE 2:** Explanation of the different GS and FSS populations and annotations of antagonists

Annotation of populations	Explanation^*a*^
GS_53_, FSS_25_	Total populations (53 and 25 isolates) of isolated antagonists
GS_48_, FSS_23_	Populations of antagonists after QC
GS_23_, FSS_23_	Representatives of the quality-controlled population of antagonists used to seed the ProbCA
GS_38_, FSS_13_	Total population of exDNase-producing antagonists after QC
GS_13_, FSS_13_	Representatives of the exDNase-producing population of antagonists used to seed the ProbCA

a QC, quality control.

The aggressiveness index (AI) was determined for every strain by taking the number of other strains antagonized by a given strain and subtracting the number of other strains antagonizing it (15).

### Estimation of coexistence using a ProbCA.

We developed a probabilistic cellular automaton (ProbCA) in Mathematica (11.3.0.0). The details of the development and code are provided in Text S4. The initial grid (350 × 350) was randomly and uniformly seeded. Each cell was chosen as the focal cell in random order and interacted with a randomly chosen neighbor cell according to the interaction matrix, and 100 simulations of 100 iterations were run.

Coexistence (richness) is the number of isolates present in the dynamic equilibrium at the end of the simulation and is used in microbiology as a measure of diversity that seems to correlate with other regularly used diversity indices. In addition, for reasons described in Text S4, the distribution of isolates in the population in the dynamic equilibrium was used to estimate Shannon diversity.

### The input matrices and simulations.

The three replicate interaction matrices were used to create a consensus binary interaction matrix using the majority rule. After quality control, five strains from the GS and two from the FSS matrices were removed for being compromised in quality (>10% of the data inconclusive or not tested) or lacking antagonist activity on any indicator, compromising the simulation. Preliminary results showed that coexistence is not necessarily a reliable measure, since coexistence increases with increasing grid size (Text S4). Due to this concern, isolates not involved in any antagonist interactions were excluded from the analysis. The forest subsurface (FSS_23_) population was composed of 23 isolates (529 tests), and the grassland surface (GS_48_) population was composed of 48 isolates (2,304 tests). To ensure a good direct comparison between simulations using the GS_48_ and FSS_23_ populations (Table 2), the simulations were first seeded using the consensus interaction matrix to determine the 23 best-surviving isolates, followed by running the simulation using these 23 isolates (Table 2). Preliminary experiments indicated that no significant differences were found in ranking and relative presence of the isolates if only this subset of isolates was reseeded and the simulations run again. A similar procedure was applied to identify 13 exDNase-producing isolates from the 38 exDNase isolates in the GS population (GS_38_) (Table 2).

### Microbial identification using a partial 16S rRNA sequence.

To determine the genera of the soil isolates, we amplified the 16S rRNA genes and determined their sequences. To amplify the 16S rRNA gene, cells from a single colony were suspended in 100 μL sterile 1× PBS using a sterile toothpick. Five microliters of this suspension was used as the template in colony PCR using Illustra PuReTaq Ready-To-Go PCR beads (catalog number 46-001-014 [Fisher Scientific]) with 1 μL of forward primer (pA, 27F, or bac8F, 20 μM, 5′-AGAGTTTGATCCTGGCTCAG-3′) (12, 52, 53), 1 μL of reverse primer (1492R, 20 μM, 5′-GGTTACCTTGTTACGACTT-3′) (53), and 23 μL water, totaling 30 μL. Primers were purchased from IDT. Amplification was achieved using the following program: 94°C for 10 min, 30 cycles of 94°C for 30 s, 58°C for 30 s, 72°C for 1 min 50 s, and finally, 72°C for 10 min. All reactions were performed in an MJ Research PTC-200 Peltier thermal cycler in the Center for Molecular Biology at Smith College. After amplification, a 5-μL sample was tested for the correct fragment size using 1.0% (wt/vol) agarose (CAS Registry Number [CAS RN] 9012-36-6; AmericaBio) gels in 1× Tris-acetate-EDTA (TAE) electrophoresis buffer with SYBR green (catalog number S3312; Invitrogen). The products were cleaned using an EdgeBio Performa DTR gel filtration cartridge (product number 42451; EdgeBio) and Sanger sequenced with the following reaction mixture and program: 1 μL BigDye (catalog number 4337454; Applied Biosystems), 5 μL double-distilled water (ddH_2_O), 0.5 μL primer (15 pM), and 3.5 μL template. The primers used were pA or 27F (5′-AGAGTTTGATCCTGGCTCAG-3′) (12, 52, 53) and 806R (5′-GGACTACHVGGGTWTCTAAT-3′) (54). The thermocycler program for labeling was 96°C for 5 min, 0.7°C/s to 96°C, 96°C for 10 s, 0.7°C/s to 50°C, 50°C for 5 s, 0.7°C/s to 60°C, 60°C for 4 min, repeat 27 times, 1°C/s to 4°C, 4°C forever. After the labeling reaction, the samples were cleaned using gel filtration and the nucleotide sequence determined using an Applied Biosystems 3130xl Genetic Analyzer in the Center for Molecular Biology at Smith College. 16S rRNA gene sequence quality control (QC) was completed using 4Peaks 1.7.1 (nucleobytes.com). Alignments of the 16S rRNA gene sequences were made in the Lasergene package SeqMan Pro (version 15.3.0 Intel), and a consensus sequence was generated from at least three sequences from at least two different PCRs and sequenced in reverse and forward directions, unless indicated otherwise. A majority rule with quality weights for consensus calling of 66% was used, and an average of 470 nucleotides/isolate was obtained. The consensus was used as the query for molecular identification using the NCBI databases. The Basic Local Alignment Search Tool was used to navigate through databases and compare nucleotide sequences from the bacterial 16S rRNA gene consensus sequences to a library of published sequences. The NCBI search option “complete genomes” was employed. The first published result with the highest percent identity, highest maximum score, and highest total score was recorded (55–57).

### Mathematical manipulations and statistical analysis.

All statistical tests confirmed the theory explained in Kanji’s *100 Statistical Tests* (58). All statistical tests involving the *t* test were executed in Excel using the one-sided, homoscedastic *t* test, unless mentioned differently. The *F* test was used to estimate significance in variation, and the Poisson test was used to estimate the significance between two observations. Furthermore, the binomial sign test and the Dixon *Q* test for outliers were performed in Excel. To determine if the interactions were equally distributed or differed from an expected population, the expected random distributions were calculated, and the observed data were tested against the expected using the χ^2^ test in Excel. PCA was done online using default settings (59).
