# Supplementary material for: Extracellular DNAses Facilitate Antagonism and Coexistence in Bacterial Competitor-Sensing Interference Competition
Source: Appl Environ Microbiol. 2022 Nov 14;88(23):e01437-22. doi: 10.1128/aem.01437-22 (PMC9746292; doi:10.1128/aem.01437-22)
Supplement: Supplemental file 1 — Supplemental material. Download aem.01437-22-s0001.pdf, PDF file, 6.5 MB [file aem.01437-22-s0001.pdf]

Supplemental Material for Ogawa et al. 2022

ExDNases facilitate Antagonism and Coexistence in Bacterial Competitor-Sensing-Interference-Competition

Aoi Ogawa[c], Christophe Golé[b], Maria Bermudez[a], Odrine Habarugira[a], Gabrielle Joslin[a], Taylor McCain[a], Autumn Mineo[a], Jennifer Wise[a], Julie Xiong[a], Katherine Yan[a], and Jan A.C. Vriezen[a,*]

Affiliations: [a] Department of Biological Sciences, 44 College Lane, Smith College, Northampton, Massachusetts 01063, USA. [b] Department of Mathematical Sciences, Smith College, Northampton, Massachusetts, USA. [c] Department of Statistics and Data Sciences, Smith College, Northampton, Massachusetts, USA.

[*]**Corresponding Author**: Jan A.C. Vriezen.
Email: jvriezen@smith.edu

This PDF file includes:
Supplemental Material:

SM#		Page#
#1	Methods to determine %antagonists.	2
#2	Basic soil edaphic characteristics	3
#3	Interaction matrices, connectance, test on TSA and DNA plates.	4
#4A	Development of a Probabilistic Cellular Automaton with code.	8
#4B	Movie Example of a Simulation	21
#5	Molecular Identification using partial 16S sequencing.	22
#6	Contribution of the third component.	23
#7	Bacterial space and relative toxin concentrations	24
#8	exDNase activity strain *Serratia* sp. CWZ222.	26

Supplemental Material #1: Methods to determine %antagonists.

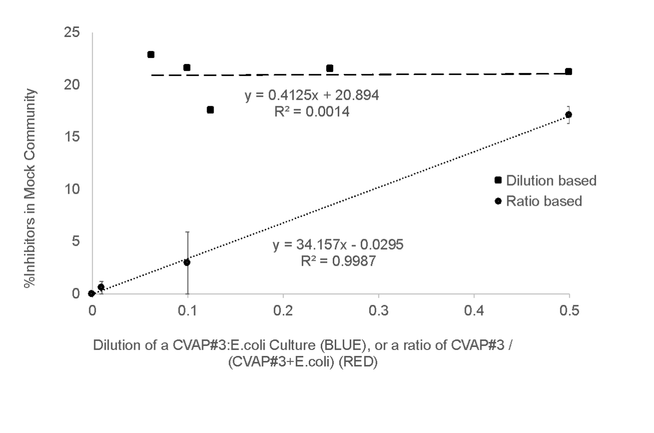

Figure S1-1: Recovery of *Pseudomonas* CVAP#3 in a mock community with *E.coli*. Black squares are a mock community of 1:1 in different densities to test the effect of dilutions. Black circles represent a mock community of 1:2, 1:10, 1:100 to test the effect of ratios.

In order to determine the relative number of antagonists in a soil population, we first tested our ability to retrieve expected ratios of antagonists in a mock community made of *E. coli* MC4100 (1) and *Pseudomonas* CVAP#3 (2). To this end, o/n cultures of *E. coli* and *Pseudomonas* CVAP#3 were grown at 25˚C in a rotator at max speed (Innova 4130). At the onset of the experiment, *E. coli* and *Pseudomonas* CVAP#3 were mixed 1:1 and diluted till a desired dilution in PBS, after with 10-fold dilution series were plated on 10% TSA. After 36h at 25˚C, colonies were counted and their ability to inhibit *Staphylococcus* CWZ226 (2) determined. As the data in Figure S2-1 indicates, we were able to retrieve the expected number of antagonist from these mock-populations since no correlation with cell-density was seen in the 1:1 co-inoculated populations. In addition, *E. coli* and *Pseudomonas* CVAP#3 were also mixed in different ratios after which 10-fold dilution series in PBS were plated on 10% TSA. After 36h at 25˚C, colonies were counted and their ability to inhibit *Staphylococcus* CWZ226 determined. Strict correlation (R^2=0.999) was observed with the different ratios of the mixed *E.coli* and *Pseudomonas* CVAP#3. These results support the validity of the method to determine the relative number of antagonists in a population.

References

1. Kanegusuku GA, Stankovic IN, Cote-Hammarlof PA, Yong PH, White-Ziegler CA. 2021. A Shift to Human Body Temperature (37°C) Rapidly Reprograms Multiple Adaptive Responses in *Escherichia coli* That Would Facilitate Niche Survival and Colonization. J Bacteriol 203:e00363-21.

2. McCall BL, Vriezen JAC. 2021. Draft Genome Sequences of *Staphylococcus* sp. Strain CWZ226, of Unknown Origin, and *Pseudomonas* sp. Strain CVAP#3, Antagonistic to Strain CWZ226. Microbiol Resour Announc 10:e00688-21.

Supplemental Material #2: Basic soil edaphic characteristics

Table S2-1: Basic soil edaphic- and climate characteristics					
		**Soil Sample**			
		**GS-3cm**	**GSS-20cm**	**FS-O horizon**	**FSS-A/B horizon**
**Temperature range (˚C)**	**Air**	20 to 24		12 to 18	
	**Soil**	15 to 16	15 to 17	13 to 15	13 to 15
**Water content %**	**Average**	20.6	16.3	36.4	16.9
	**Count (n=)**	9	9	9	9
	**SEM**	0.97	0.57	2.41	0.98
**%N**	**Average**	0.34	0.25	0.95	0.27
	**SEM**	0.02	0.02	0.10	0.02
	**n=**	3	3	3	2
**%C**	**Average**	4.32	3.29	24.52	7.33
	**SEM**	0.30	0.22	2.29	0.78
	**n=**	3	3	3	2
**C/N**	**Average**	12.6	13.0	25.9	27.2
	**SEM**	0.4	0.2	1.0	1.6
	**n=**	3	3	3	2
**pH**	**Average**	6.2	6.1	4.3	5.0
	**SEM**	0.06	0.03	0.20	0.00
	**n=**	3	3	3	3
**CFU/g dry soil**	**Average**	4.41×10^6	3.12×10^6	1.68×10^6	5.01×10^5
	**SEM**	2.6×10^5	2.8×10^5	7.3×10^5	1.0×10^4
	**n=**	3	3	3	3
**% Antagonists**	**Total screened**	517	494	492	462
	**# Antagonists**	53	46	28	25
	**% Antagonists**	10.3	9.3	5.7	5.4

Supplemental Material #3:

This supplemental material contains the pair wise test for antagonism to determine the connectence (Table S3-1 for GS_{48}, and Table S3-2 FSS_{23}). Furthermore, this supplemental material also contains the response of isolates to a decrease in nutrients and exDNase production (Table S3-3). Development of a Zone Of Inhibition (ZOI) is indicated with a “+”, no ZOI is indicated with a “-“. Missing data is indicated with “ND”.

Table S3-1: Pairwise interaction matrix for the GS_{48} population. Reported is the consensus.

TOXIN PRODUCING ISOLATES	INDICATOR STRAINS																																												
CVAP#	401	402	403	404	406	407	451	453	454	457	458	459	460	461	462	463	464	542	544	546	553	559	563	691	695	698	703	707	710	721	726	727	728	729	736	737	738	739	740	741	742	743	746	748	750
401	-	-	-	-	-	-	+	+	+	+	-	-	-	-	-	-	-	-	-	-	-	+	+	+	+	-	-	+	+	-	-	+	-	-	-	-	-	-	-	+	-	-	+	-	-
402	+	-	-	+	+	-	+	+	+	-	+	+	-	-	-	-	+	+	+	-	+	-	-	+	+	+	+	-	-	-	-	-	-	+	+	+	-	-	+	+	-	-	+	-	-
403	+	-	-	-	-	+	-	-	-	-	+	+	-	+	-	+	-	-	-	-	-	-	-	-	+	+	-	-	+	+	-	+	+	+	+	-	-	-	+	-	+	-	+	+	+
404	+	-	-	-	-	-	+	+	+	+	+	+	+	-	+	+	+	-	-	-	-	-	-	+	+	-	+	+	-	-	-	-	-	-	-	-	-	-	+	+	-	+	+	-	-
406	+	-	-	-	-	-	+	+	+	-	-	+	-	-	-	-	+	-	-	-	-	-	+	ND	+	-	-	+	-	-	-	+	-	-	-	-	-	-	+	+	-	-	+	-	-
407	+	-	-	-	-	-	+	-	-	+	+	-	-	-	-	-	+	+	+	-	-	+	-	ND	-	-	-	-	+	-	-	+	-	-	+	-	-	-	-	+	-	-	-	-	-
451	+	+	-	+	+	-	-	-	-	+	+	-	-	-	-	-	+	-	-	+	-	+	+	-	-	+	+	-	-	-	-	+	+	+	+	+	+	-	+	+	+	+	-	+	-
453	-	-	ND	-	+	-	+	-	-	-	-	+	-	-	-	-	-	-	-	-	-	-	-	-	+	-	-	-	-	-	-	+	-	-	-	-	-	-	-	-	-	-	-	+	-
454	+	+	-	+	+	+	-	-	-	+	+	-	-	-	-	-	+	-	-	+	-	+	+	-	-	+	+	-	-	-	+	+	+	ND	+	+	+	-	+	+	+	+	-	+	+
457	+	-	-	+	+	-	+	+	+	-	+	+	-	+	-	-	+	-	+	+	+	-	-	+	+	+	+	+	+	-	-	+	+	-	+	+	+	+	+	+	+	+	+	+	+
458	-	+	-	+	+	-	-	-	-	-	-	-	-	-	-	-	-	-	-	-	+	+	+	-	+	+	-	+	+	-	+	+	+	+	+	+	+	+	+	-	+	-	+	+	-
459	+	+	+	+	+	-	-	-	-	+	-	-	-	-	-	+	+	-	-	-	-	-	-	-	-	+	-	-	-	+	+	-	+	-	+	+	-	-	-	-	+	-	-	-	-
460	-	-	+	+	-	-	-	-	+	-	+	+	-	-	-	-	+	-	+	+	+	-	-	-	-	-	-	-	-	+	-	-	-	-	-	-	+	-	-	-	+	-	+	+	-
461	+	+	-	+	+	-	+	-	+	+	+	-	-	-	+	-	+	+	+	+	+	+	+	+	+	+	+	+	+	+	+	+	+	+	+	+	-	-	+	+	+	+	+	+	+
462	-	-	-	-	-	-	-	-	-	+	+	-	-	-	-	-	+	+	+	+	+	+	+	-	+	-	-	+	+	+	-	+	-	-	+	+	+	-	+	-	-	+	-	-	+
463	+	+	-	+	+	-	+	-	+	+	+	-	-	-	+	-	+	+	+	+	+	-	+	+	+	+	+	-	+	+	+	+	+	+	+	+	-	+	+	+	+	+	+	+	+
464	-	-	-	-	-	-	-	-	-	-	-	+	-	-	-	-	-	-	-	-	-	-	-	-	-	-	-	-	-	-	-	-	-	-	-	-	-	-	-	-	-	-	-	-	-
542	-	-	-	-	-	-	-	-	-	-	-	-	-	-	-	-	-	-	-	-	-	-	-	-	-	-	-	-	-	-	-	-	-	-	-	-	+	-	-	-	+	-	-	-	+
544	-	-	-	-	-	-	-	-	+	-	-	-	-	-	-	-	-	-	-	-	-	-	-	+	-	-	-	-	-	-	-	-	-	-	-	-	-	-	-	-	-	-	-	-	-
546	-	+	-	-	+	-	-	-	-	-	-	-	-	-	-	-	-	-	-	-	+	-	+	-	-	+	+	+	-	-	-	-	-	-	-	-	+	-	-	-	+	-	-	+	-
553	-	-	-	-	-	-	+	-	+	-	-	-	-	-	-	-	-	-	-	-	-	-	-	+	+	-	-	+	-	-	-	+	-	-	-	-	-	-	+	+	-	-	+	-	-
559	+	-	-	+	+	+	+	-	+	-	+	-	-	-	-	-	+	+	+	-	+	-	-	+	+	+	+	+	+	+	-	-	+	+	+	+	+	+	+	-	+	-	+	-	-
563	+	-	-	+	+	+	+	-	+	-	+	-	-	+	-	-	+	+	+	+	-	-	-	+	+	+	+	+	+	+	-	+	+	+	+	+	+	+	+	+	+	+	+	+	-
691	+	+	-	+	+	-	-	-	-	+	+	-	-	-	-	-	+	+	-	+	+	+	+	-	-	+	+	+	+	+	+	+	+	+	+	+	+	-	+	+	+	+	-	+	+
695	+	+	-	+	+	-	+	+	+	+	+	+	-	-	-	-	+	-	-	+	+	+	+	-	-	+	+	+	+	+	+	+	+	+	+	+	+	+	+	+	+	+	-	+	+
698	-	-	-	-	-	-	+	+	+	-	-	+	-	-	+	-	+	-	-	+	-	+	-	+	+	-	-	+	-	-	-	+	-	-	-	-	-	-	+	+	-	+	+	-	-
703	+	-	-	-	-	-	+	-	+	-	+	+	-	-	-	-	+	-	-	-	-	-	-	+	+	-	-	-	-	+	-	-	-	-	-	-	-	-	-	+	-	-	+	-	-
707	+	+	-	+	+	-	-	-	-	+	+	-	-	-	-	-	-	-	+	-	+	+	+	-	-	+	-	-	-	-	+	-	+	+	+	-	-	-	-	-	+	-	-	+	-
710	+	+	-	+	-	-	+	-	+	+	-	+	-	-	-	-	+	-	-	+	-	+	+	-	-	+	+	+	-	-	+	+	+	+	+	+	+	+	+	+	+	+	-	+	-
721	+	+	-	+	+	-	+	-	-	+	-	-	-	-	-	-	+	-	-	+	+	+	+	+	-	+	-	-	+	-	-	+	+	+	+	+	-	-	+	-	+	-	-	+	+
726	-	-	-	-	-	-	+	+	+	-	-	+	+	-	-	+	+	-	-	+	-	-	-	+	+	-	+	-	-	-	-	-	-	-	-	-	-	-	+	+	-	+	+	-	-
727	-	+	-	-	+	-	+	-	-	+	+	-	-	-	-	-	-	-	-	-	+	+	+	+	+	+	+	+	-	-	+	-	+	-	-	+	-	-	-	-	-	-	+	+	-
728	-	-	-	-	-	-	+	+	+	+	-	+	-	-	-	-	+	-	-	+	-	-	-	+	+	-	-	+	-	-	-	-	-	-	-	-	-	-	+	+	-	-	+	-	-
729	-	+	-	-	-	-	+	+	+	+	-	+	-	-	-	-	+	-	-	-	-	-	-	+	+	-	+	+	-	-	-	-	-	-	-	-	-	-	+	+	-	-	+	-	-
736	-	+	-	-	-	-	+	+	+	+	-	+	-	-	-	-	+	-	-	-	-	-	-	+	+	-	+	-	-	-	-	-	-	-	-	-	-	-	+	+	-	+	+	-	-
737	-	-	-	-	-	-	+	-	+	+	-	+	-	-	-	-	+	-	-	-	-	-	-	+	+	-	-	+	-	-	-	-	-	-	-	-	-	-	+	+	-	-	+	-	-
738	-	-	-	-	-	-	+	-	+	-	-	-	-	-	-	-	-	-	-	+	-	+	-	+	+	-	-	-	-	-	-	-	-	-	-	-	-	-	-	-	-	-	+	-	-
739	-	-	-	-	-	-	-	-	-	+	-	-	-	-	-	-	+	-	-	-	-	-	+	-	+	-	-	-	-	-	-	-	-	-	-	-	-	-	-	+	-	-	-	-	-
740	-	-	-	-	-	-	-	-	-	+	+	-	-	-	-	-	-	-	-	-	+	-	+	-	-	+	-	-	-	-	-	-	-	-	-	-	-	-	-	+	+	-	-	+	-
741	+	-	-	+	+	-	+	-	+	+	-	-	-	-	-	-	-	-	-	-	-	+	+	+	+	+	-	-	-	-	-	-	+	+	+	-	-	-	-	-	+	+	+	+	-
742	-	-	-	-	-	-	+	+	+	-	-	+	+	-	-	-	+	-	-	-	-	-	-	-	+	-	-	+	-	-	-	-	-	-	-	-	-	-	+	+	-	-	+	-	-
743	+	-	-	-	-	-	+	-	+	+	+	+	-	-	-	-	+	-	-	+	-	-	+	-	+	-	-	-	+	-	+	-	-	-	-	-	-	-	-	+	-	-	+	-	+
746	+	+	-	+	+	+	+	-	+	+	+	-	-	ND	-	-	+	-	-	+	+	+	+	-	-	+	+	+	+	+	+	+	+	+	+	+	+	+	+	+	+	+	-	+	+
748	-	+	-	-	-	-	-	+	+	-	-	-	-	-	-	-	+	-	-	+	-	-	-	+	+	-	+	+	-	-	-	+	-	-	-	-	+	-	+	+	-	-	+	-	-
750	-	-	-	-	-	-	+	-	-	-	-	-	-	ND	-	-	+	-	-	+	-	+	+	-	-	-	+	-	-	-	-	-	-	-	-	-	-	-	-	+	-	-	+	-	-
762	+	+	-	+	+	-	+	-	+	+	+	-	-	-	-	-	+	-	-	+	+	+	+	-	-	+	+	+	-	+	+	+	+	+	+	+	+	+	+	+	+	+	-	+	+
764	+	-	-	-	-	-	+	+	+	-	-	-	-	-	+	-	+	-	-	+	-	-	-	+	+	-	+	+	+	-	-	+	-	-	-	-	+	+	+	+	-	+	+	-	-
767	+	-	-	+	+	+	+	-	+	-	+	-	-	ND	-	-	+	+	+	+	+	-	-	+	+	+	+	+	+	+	-	+	+	+	+	+	+	+	+	+	+	+	+	+	+

Removed were CVAP# 452, 541, 550, 558 for too many missing data. CVAP456 is too inert and does not produce/respond

ND = Not Determined

The consensus is based on the majority rule for three independent determinations

+ = ZOI, - = No ZOI

Table S3-2: Pairwise interaction matrix for the FSS_{23} population. Reported is the consensus.

		INDICATOR STRAINS																						
	CVAP#	433	434	435	436	438	439	440	531	534	535	621	623	630	654	656	658	659	660	662	663	666	781	782
TOXIN PRODUCING ISOLATES	433	-	-	+	-	-	-	-	-	-	-	-	+	-	-	+	-	+	-	-	-	-	+	+
	434	-	-	-	-	-	-	-	-	-	-	+	+	-	-	-	+	-	-	-	-	-	+	-
	435	-	-	-	+	-	-	-	+	-	-	+	+	-	-	+	-	+	+	+	+	-	-	-
	436	-	-	-	-	-	-	-	-	-	-	-	-	-	-	-	-	-	-	+	-	-	-	-
	438	-	+	+	-	-	-	+	+	-	+	+	+	-	-	-	+	+	-	-	-	-	+	+
	439	-	-	+	-	-	-	+	+	-	+	+	+	+	+	+	+	+	+	+	+	-	+	-
	440	-	-	-	-	-	-	-	-	-	-	+	-	-	-	-	-	+	+	-	-	-	-	-
	531	-	-	-	-	-	-	-	-	-	-	-	+	-	-	+	-	-	+	-	+	-	-	+
	534	-	-	-	-	-	-	-	-	-	-	+	-	-	-	-	-	-	+	-	+	-	-	-
	535	-	-	-	+	-	-	-	+	-	-	+	+	-	-	+	-	+	+	-	+	-	-	-
	621	-	-	+	-	-	-	-	-	-	-	+	+	-	-	-	+	-	-	+	-	-	-	-
	623	-	+	-	-	-	-	-	-	-	-	-	-	-	-	-	-	-	-	-	-	-	-	-
	630	-	+	-	-	-	-	+	-	+	+	+	+	-	+	-	-	+	+	+	+	-	-	-
	654	-	-	+	-	-	-	+	+	-	+	-	-	+	-	+	+	+	-	+	+	-	+	+
	656	-	-	+	-	-	-	+	-	+	+	-	+	-	-	-	-	-	+	+	+	-	-	-
	658	-	-	-	-	-	-	+	-	-	+	+	+	-	-	+	+	-	+	+	-	-	-	+
	659	-	-	+	-	-	-	-	+	-	+	+	+	-	-	+	+	-	+	-	+	-	+	+
	660	-	-	-	+	-	-	+	-	-	-	+	+	-	-	+	-	+	-	+	+	+	+	+
	662	-	-	+	+	-	-	+	+	+	+	+	+	-	-	+	+	+	+	-	+	+	+	+
	663	-	-	+	+	-	-	+	+	-	+	+	+	+	-	-	+	+	+	+	-	+	+	+
	666	-	-	-	-	-	-	-	-	-	+	-	-	-	-	-	-	-	+	-	-	-	-	-
	781	-	-	-	+	-	-	-	+	-	-	+	+	-	-	+	-	+	+	-	+	+	-	-
	782	-	-	-	+	-	-	-	+	-	-	+	+	-	-	+	-	+	+	-	+	+	-	-

CVAP533 was removed since it contained too many undetermined data. CVAP441 was removed for being too inert.

The consensus is based on the majority rule for three independent determinations

+ = ZOI, - = No ZOI

Table S3-3: Phenotypic data from GS_{48} and FSS_{23} populations

					Nutrient Experiment			Complementation					On DNA plates	
CVAP#	Soil Source	# strains it antagonizes	# strains it is antagonized by	AI	10% TSA	50% TSA	100% TSA	10% TSA + 100mM Glucose	10% TSA + 120mM Ribose	10% TSA + 200mM Pyruvate	10% TSA + 50mM NH4NO3	100mM = K3PO4	ZOI on Dnase plates	exDNase activity
**401**	**GS**	14	25	-11	-	-	-	-	+	-	-	-	+	+
**402**	**GS**	24	18	6	-	+	+	-	-	+	-	-	+	+
**403**	**GS**	20	2	18	-	-	-	-	-	-	-	-	+	+
**404**	**GS**	20	20	0	-	+	+	+	+	-	-	-	+	+
**406**	**GS**	14	21	-7	-	+	+	+	+	-	-	-	+	+
**407**	**GS**	12	6	6	-	+	-	-	+	-	-	-	-	+
**433**	**FSS**	6	0	6	+	+	+	+	+	+	+	+	-	-
**434**	**FSS**	4	3	1	+	+	+	+	+	ND	+	+	-	-
**435**	**FSS**	9	9	0	+	+	+	+	+	+	+	+	+	+
**436**	**FSS**	1	7	-6	-	-	-	+	+	-	-	+	+	+
**438**	**FSS**	11	0	11	-	-	-	+	-	+	+	-	-	-
**439**	**FSS**	15	0	15	+	+	+	+	-	+	+	+	+	+
**440**	**FSS**	3	9	-6	+	-	-	+	+	+	+	-	+	+
**441**	**FSS**	0	0	0	-	-	-	+	+	-	+	ND	-	-
**451**	**GS**	25	35	-10	-	-	+	+	ND	-	-	ND	+	+
**453**	**GS**	7	14	-7	-	-	+	+	+	-	-	-	+	-
**454**	**GS**	26	30	-4	-	-	-	+	ND	ND	-	-	+	+
**457**	**GS**	33	25	8	-	+	+	+	+	-	-	-	+	+
**458**	**GS**	25	23	2	-	-	-	-	+	-	-	-	+	+
**459**	**GS**	16	19	-3	-	+	+	-	ND	-	-	+	-	-
**460**	**GS**	15	3	12	+	-	+	+	ND	-	-	-	-	-
**461**	**GS**	39	3	36	+	+	-	+	+	-	-	-	+	+
**462**	**GS**	20	5	15	+	-	+	+	+	-	-	-	-	-
**463**	**GS**	38	3	35	-	+	-	+	+	-	-	-	+	+
**464**	**GS**	1	34	-33	-	+	+	+	+	ND	-	+	-	-
**531**	**FSS**	5	10	-5	+	+	+	+	+	-	+	-	+	+
**535**	**FSS**	8	10	-2	+	+	+	+	+	-	+	-	+	+
**542**	**GS**	3	9	-6	-	-	-	+	+	-	+	-	-	-
**544**	**GS**	2	11	-9	+	-	-	+	+	-	+	-	-	-
**546**	**GS**	10	23	-13	-	-	-	-	+	-	-	-	+	+
**553**	**GS**	11	18	-7	-	-	-	-	+	-	-	-	+	+
**559**	**GS**	29	19	10	-	+	+	+	ND	-	-	-	+	+
**563**	**GS**	34	22	12	-	+	-	+	+	+	-	-	+	+
**621**	**FSS**	5	16	-11	-	-	-	-	-	-	-	-	+	+
**623**	**FSS**	1	17	-16	+	-	-	-	-	-	-	-	-	-
**630**	**FSS**	11	3	8	-	+	+	+	+	+	+	+	-	-
**654**	**FSS**	12	2	10	-	-	-	-	+	ND	+	+	-	-
**656**	**FSS**	8	12	-4	-	+	+	-	+	-	-	-	-	-
**658**	**FSS**	9	9	0	-	+	-	-	-	ND	-	-	-	-
**659**	**FSS**	11	13	-2	-	+	+	-	-	-	-	-	+	+
**660**	**FSS**	11	15	-4	+	ND	+	-	+	+	+	+	+	+
**662**	**FSS**	16	10	6	+	+	+	+	+	+	+	+	+	+
**663**	**FSS**	15	13	2	+	+	+	+	+	ND	+	+	-	+
**666**	**FSS**	2	5	-3	-	+	+	+	-	-	-	-	-	-
**691**	**GS**	32	24	8	-	-	+	+	+	-	-	-	+	+
**695**	**GS**	36	30	6	-	-	+	+	+	-	-	-	+	+
**698**	**GS**	17	23	-6	+	+	+	+	+	ND	-	-	+	+
**703**	**GS**	11	23	-12	-	-	-	-	+	-	-	-	+	+
**707**	**GS**	18	25	-7	-	-	-	+	+	ND	-	-	+	+
**710**	**GS**	29	16	13	-	+	+	-	+	-	-	+	+	+
**721**	**GS**	26	15	11	-	+	+	+	+	-	-	-	+	+
**726**	**GS**	16	12	4	+	+	+	-	+	-	+	-	+	+
**727**	**GS**	20	25	-5	-	-	-	+	+	-	-	-	-	-
**728**	**GS**	14	20	-6	-	+	+	-	+	-	-	-	+	+
**729**	**GS**	15	17	-2	-	+	+	-	+	-	+	-	+	+
**736**	**GS**	15	22	-7	-	+	+	-	+	-	-	-	+	+
**737**	**GS**	12	19	-7	-	+	+	-	+	ND	-	-	+	+
**738**	**GS**	7	18	-11	-	-	-	-	+	-	-	-	+	-
**739**	**GS**	5	11	-6	-	-	-	+	+	-	-	-	+	+
**740**	**GS**	9	30	-21	-	-	-	+	+	+	-	-	+	+
**741**	**GS**	19	32	-13	-	-	-	-	+	-	-	-	+	+
**742**	**GS**	12	23	-11	-	+	+	+	+	ND	-	-	+	+
**743**	**GS**	16	19	-3	-	-	+	-	+	-	-	-	-	-
**746**	**GS**	35	29	6	-	+	+	+	-	-	-	-	+	+
**748**	**GS**	15	22	-7	-	+	+	+	ND	-	+	-	+	+
**750**	**GS**	9	14	-5	-	-	+	+	-	ND	-	-	-	+
**762**	**GS**	33	28	5	-	-	+	+	-	-	-	-	+	+
**764**	**GS**	20	22	-2	-	+	+	-	+	ND	-	+	+	+
**767**	**GS**	35	10	25	-	+	+	-	+	ND	-	-	+	+
**781**	**FSS**	9	9	0	-	+	+	+	+	-	-	-	+	+
**782**	**FSS**	9	9	0	-	+	+	-	+	-	-	-	+	+

ND = Not Determined

The consensus is based on the majority rule for three independent determinations

+ = ZOI, - = No ZOI

Supplemental Material #4A: Development of a Probabilistic Cellular Automaton

Introduction

A Cellular Automaton (CA) consists of a toroidal regular planar grid of virtual-cells which evolves through discrete time steps and discrete states, according to rules based on the state of neighboring cells. If the rule is probabilistic, as in our case, the model is a Probabilistic Cellular Automaton (ProbCA) in which a randomly chosen neighbor cell will replace the focal cell according to conditions defined in an interaction matrix. A (Probabilistic) Cellular Automaton is a model useful for simulating complex and nonlinear dynamics and is used in studies to understand bacterial communities. For example, *in-vitro* and *in-vivo* game-theoretical dynamics among three phenotypes of bacteria: killer (aggressor), resistant, and sensitive, lead to a stable dynamic equilibrium in the stationary phase and was modeled using a cellular automaton (1,2,3,4).

Cellular automata can also help to study coexistence in a dynamic equilibrium and are thus employed to explore the effect of bacterial interactions on biodiversity (5,6,7). For example, using bacterial populations derived from soil, Abrudan (6) used species observed (Richness) as an index to measure the coexistence among *Streptomyces*. In another study, using isolates from water, an environment less structured than soil, Zapien-Campos (7) found that more aggressive isolates were more likely to occupy the cells in the dynamic equilibrium.

In order to study the effect of antagonist populations from different soils on coexistence, we first developed a Probabilistic Cellular Automaton in Mathematica (11.3.0.0) to explore its use in modeling antagonist interactions within a population of soil derived- bacterial isolates. Mathematica is used in modelling of bacterial interactions (8). Our cellular automaton is an amended version of the model created by Abrudan (6), and we tested its robustness with our experimental data in relation to the following questions: (i) How does changing the grid size affect the outcome? (ii) Is richness to estimate diversity in the dynamic equilibrium a good index? Finally, (iii) does spatial configurations affect the diversity of soil bacteria?

Results and Discussion

Table S4-1: Determination of the consensus in this study. A “1 or +” means that a zone of inhibition (ZOI) was observed on the antagonized by the antagonist. A “0 or -” means that such a ZOI was not observed.

Observation in trials 1, 2 and 3	In “consensus” interaction matrix
+, +, +	1
+, +, -	1
+, -, -	0
-, -, -	0
+, -	undetermined

The interaction matrix:
As mentioned, our ProbCA model of microbial interactions is inspired by the one used by Abrudan (6). The main differences are that we (i) only consider asocial interactions, and no social interactions, (ii) used a randomly seeded homologous ethno-sphere, (ii) of 350x350 cells in size, (iii) a randomly choose the focal cell as well as the neighboring cells, (iv) 100 iterations and (v) 100 simulations in parallel. Furthermore, we (vi) use two populations of antagonists from two different types of soil of different size. The Forest SubSurface (FSS) population was composed of 25 isolates and the Grassland Surface (GS) population was composed of 53 isolates.

In addition, (vii) based on three experimental results, binary interaction matrices were created using a consensus majority rule based on the three observations (Table S4-1) and rather than the four observations employed by Abrudan et al. (2015).
All isolates from a single source were tested pairwise against all isolates from the same source and the ability to produce a zone of inhibition (ZOI) determined (Figure S4-2A). Development of a ZOI is an indicator of the production of a toxin by the antagonist inhibiting the indicator (or antagonized). There were a total of 2809 tests for the GS population and 625 tests for the FSS population and these tests were repeated three times.

The Probabilistic Cellular Automaton:

For each population we created an interaction matrix as depicted in Figure S4-2A. This interaction matrix was used as input in the simulations. For the simulation, we seeded a square grid randomly with numbers 1 to 25 representing the FSS isolates in a homogenous ethno-sphere (Figure S4-2C). Every cell on the grid represents an isolate. Every cell was then chosen, in a random order, as a focal cell. One of the eight neighbors of the focal cell was randomly chosen, and if according to the interaction matrix the isolate represented by the neighboring cell produces a ZOI on that of the focal cell, it replaces the focal cell (Figure S4-3B). Otherwise both cells retained their isolate. Pseudocode directing this algorithm in Mathematica is provided in Figure S4-3. Iteration of this process results in the evolution of a dynamic equilibrium (Figure S4-2C). Like Zapiens-Campos (7), we observed convergence to an equilibrium in all our simulations, as well as clustering of the dominant isolates. We did not quantify either of these observations.

A

Lawn / Isolate	Bacteria A	Bacteria B	Bacteria C	Bacteria D
Bacteria A	0	1	1	0
Bacteria B	0	0	1	0
Bacteria C	1	0	0	0
Bacteria D	1	0	1	0

B

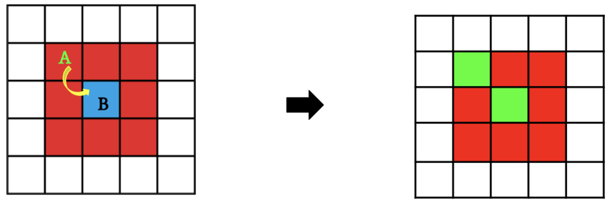

C

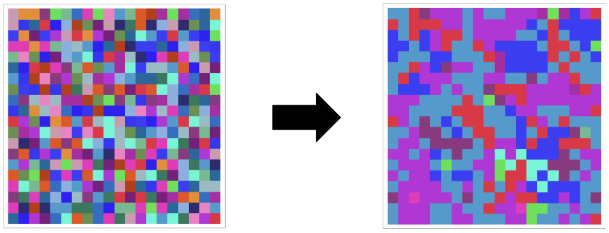

Figure S4-2: (A) Example of an interaction matrix. The isolate represents the antagonist, the “lawn” is the seeded agar plate with the antagonized isolate. A “1” means that a zone of inhibition (ZOI) was observed on the antagonized by the antagonist. A “0” means that such a ZOI was not observed. (B) An example of a replacement rule. A focal cell was assigned a random neighboring cell which after applying the “replacement rule” takes over the focal cell. The selection of the focal cell and replacement are repeated until all the 200 by 200 cells were assigned as a focal cell. The simulation ran 100 iterations with 100 replicates. (C) An example of the simulation run (right: initial condition=random and homogeneous, left: final condition=heterogenous). See Also the movie in S#4B.

Effect of grid size on the richness of final distribution

Even though Abrudan et al. (6) and Zapiens-Campos et al (7) tested the effect of (a)social interactions of *Streptomyces* and bacterial isolates from a superficial sediment on coexistence respectively, these authors did not report the effect of different matrix sizes on the final outcome. To test if the grid size affects richness in the dynamic equilibrium, the isolates were used to seed grid sizes of 20x20, 200x200, 350x350 and 500x500, and simulations were run as described earlier.

```
algorithm fielddayFSS is
    input: FSSspecies (the initial grid)
    output: updated grid with some replacements of cell according to the interaction matrix

    bigFSS ← an array which passed in grid padded with cyclic repetition of the complete
            array on the edges

    Randomrow ← randomly selected row
    Randomcolumn ← randomly selected column

    for each Randomrow in bigFSS do
        for each Randomcolumn in bigFSS do
            centerIsolate ← randomly chosen cell in bigFSS
            neighborIsolate ← randomly chose one neighbor cell from eight neighbor
                              cell surrounding centerIsolate in bigFSS
            qcenterIsolate ← get data from interaction matrix where
                              isolate =  centerIsolate and lawn = neighborIsolate
            qneighborIsolate ← get data from interaction matrix where
                              isolate =  neighborIsolate and lawn = centerIsolate
            If qcenterIsolate is smaller than qneighborIsolate
                Replace the centerIsolate with neighborIsolate
    return updated grid
```

Figure S4-3: Pseudocode for the FSS population. BigFSS refers to the toroidal shape of the grid in which the rightmost column of FSS is added to the left, the leftmost to the right, and likewise for top and bottom.

The number of isolates observed in the dynamic equilibrium were low in both Forest Subsurface (FSS) isolates and Grassland Surface (GS) isolates at grid size 20, and were 11.6 and 10.6 respectively. However, as the grid size increases an increase in richness was observed (Figure S4-4), which plateaued and reached a maximum richness at higher grid sizes. This equilibrium point for the FSS simulation was 24.1 ± 0.2 and the GS simulation results show that it has not reached maximum richness in a dynamic equilibrium yet. Due to this grid-size dependence, we conclude that richness alone is not a reliable parameter to estimate the diversity in the dynamic equilibrium. The reason beyond the richness increases with an increase in grid size can be explained by (i) when the isolates interact on a smaller grid, there is a higher probability of an isolate being eliminated by an antagonist than in a larger grid. This leads to fewer species present in a smaller grid than in a larger grid; (ii) in a larger grid size, the grid may reach a configuration where the sensitive isolate is surrounded by neutral isolates (resistant) and neutral isolates shields attack from antagonists. Moreover, some authors from *in-situ* studies of microbial diversity mentioned that richness is not a reliable measurement of diversity due to a downward-biased estimator for total species richness and lack of accuracy in measurement (11). In a small sample size, it is hard to observe all species and the species not observed can be under-sampled, thus the richness is overestimated (12). If this also applies to *in-silico* simulations remains to be seen, but based on these studies and our empirical results, we concluded not to use richness as a measurement for diversity in the dynamic equilibrium alone and thus must be supported

by a distribution measure such as Shannon’s diversity or evenness.

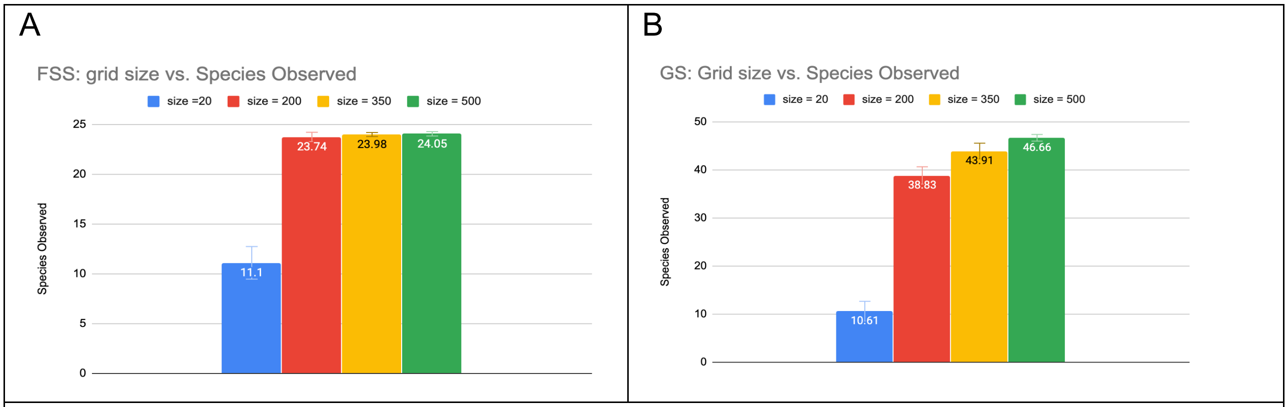

Figure S4-4: The relationship between grid sizes and species observed in (A) the FSS simulation, and (B) the GS population.

Effect of Grid Sizes on Distribution of the Resulting Community

Because richness alone is not a good predictor of diversity after employing cellular automata, the distribution of the resulting community as a measure for diversity was explored. The prediction is that despite the differences in richness in the dynamic equilibrium, the obtained community structure expressed in frequency would be constant at dynamic equilibrium across different grid sizes. The final frequency distribution was determined at the end of each replication and the mean of the distribution of 100 replicates was calculated. Spearman rank and the Chi^2 test were employed to compare the difference of the frequencies and the rank distributions. The Spearman-rank test indicates that all grid-sizes for GS (rho>0.95, $P<3x10^{-16}$) and FSS (rho>0.96, $P<3x10^{-7}$) give the same distributions based on rank (Table S4-Appendix 1A).

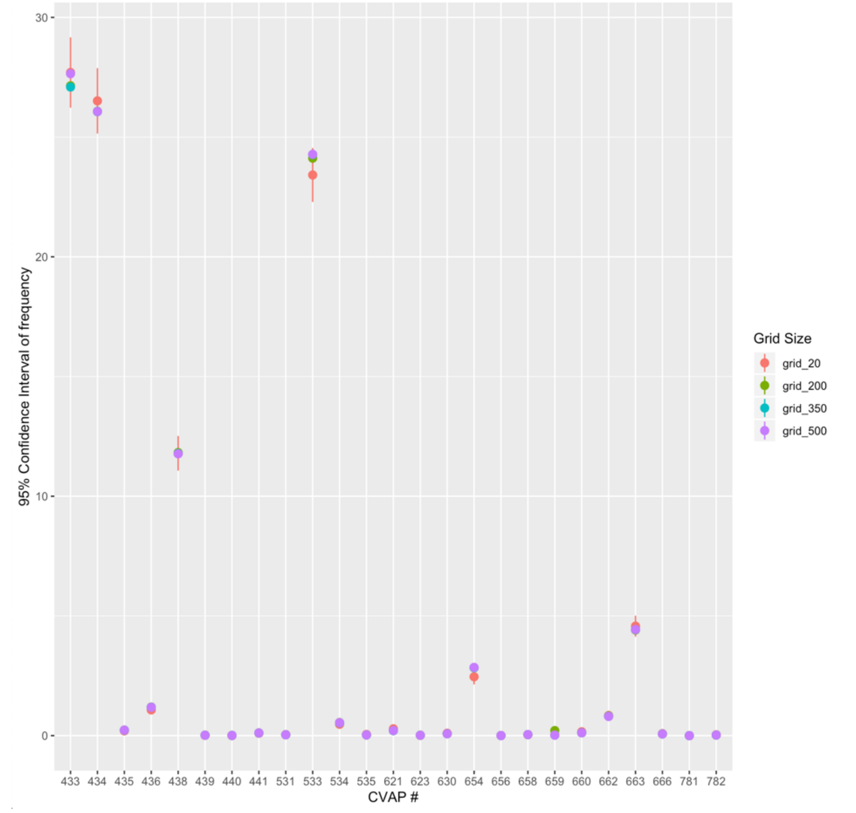

Figure S4-5: Example of the several frequency distributions obtained when simulating at different grid sizes. 95% Confidence interval of frequencies of different grid size in FSS simulations.

The Chi^2 test however, has a better ability to detect differences between the frequencies within the distributions. This resulted in a grid-size dependent frequency distribution for the GS populations (0.01<P<0.07) and FSS population (0.23<P<0.25, Table S4-Appendix 1B). The data in Figure S4-5 illustrates these findings. We conclude that for this type of experiment, the Chi^2 test is a too sensitive statistical approach. Spearman, however, suffices.

The Effect of Spatial Configurations on the Community Profile in the Dynamic Equilibrium

We observed convergence to stationary states of our Cellular Automaton after which the cell's spatial configuration no longer changes. Intuitively, this is because the vulnerable strains are shielded by strains that are resistant to the aggressive strain and non-antagonistic towards the vulnerable strains so that the aggressive strains cannot attack the vulnerable strains (7). As in that work, we checked whether the spatial distribution of the populations had an influence on the equilibrium frequency. We did so by iterating our ProbCA until it reached an equilibrium. Usually these equilibria showed substantially clustered populations of dominant isolates (See Figure S4-6). We then randomly scrambled the equilibrium and reran a number of iterations on it, resulting in a new equilibrium. In the case of the FSS, the new equilibrium showed little difference with the scrambled equilibrium, but the dominant isolates increased slightly in numbers, the weaker ones decreased, indicating that the clustering may protect some of the weaker isolates. This effect was substantially stronger for the GS populations. To determine whether the initial spatial configuration of our populations affect the community profile of isolates, we performed statistics on repeated simulations. The final distribution of the consensus experiment using the 350 by 350 grid was utilized and was reseeded randomly for a second round of simulations as shown in Figure S4-6.

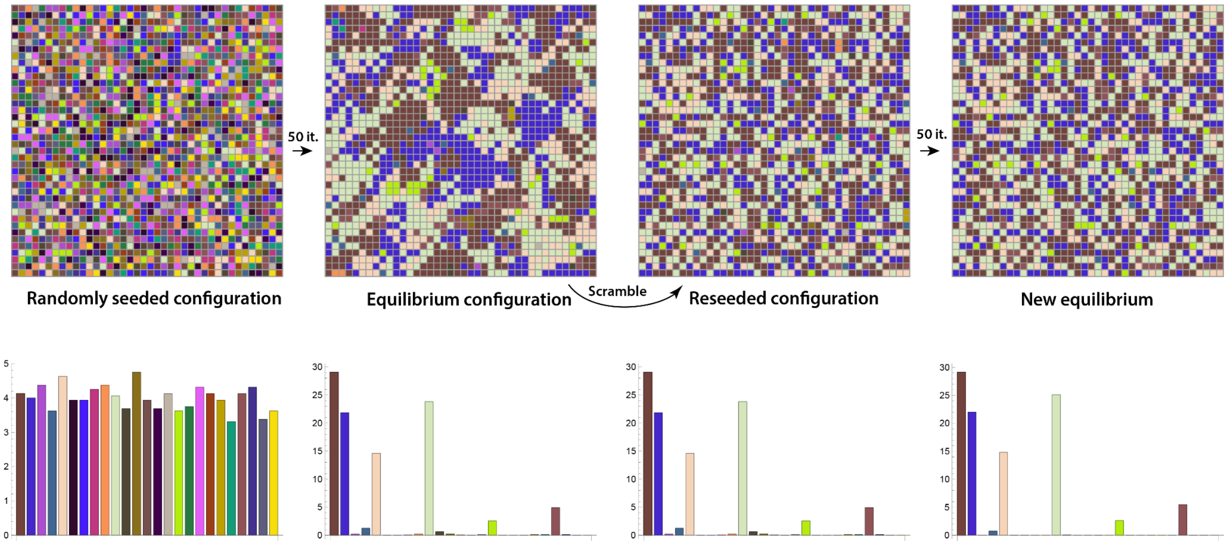

Figure S4-6: Declustering an equilibrium and iterating only changes the distribution slightly in FSS. Each bar graph corresponds to the frequency distribution of the isolates in the configuration above it. Left: Randomly seeded initial configuration. Middle Left: Equilibrium after 50 iterations, showing clustering of dominant isolates. Middle Right: the equilibrium configuration was randomly scrambled, keeping the same frequency distribution. Clustering is decreased. Right: after 50 iterations on the scrambled equilibrium, a new equilibrium emerges, retaining most features of the scrambled equilibrium. However, the frequencies of the dominant isolates have slightly increased, while those of the weaker ones correspondingly decreased. In general, we expect a decrease in richness as some isolates disappear.

The reseeded final distribution was compared with the final distribution of the first simulation. It was hypothesized that the community profile would change due to changing the position of isolates which prevents them from shielding and clustering. The Spearman rank test suggests that both soil populations had a strong correlation between the two final distributions (rho = 0.999 for the FSS simulation and rho = 0.98 for the GS simulation) and the observed correlation was not due to chance (FSS: $P=3.3\times10^{-7}$, GS: $P=3.2\times10^{-7}$). However, there was a decrease in richness in both GS and FSS cases (GS ~4 and FSS ~2). The Chi^2 result showed that for the FSS population the difference was not significant. However, for the GS population there was a significant difference between the distributions (FSS: P=0.237, GS: $P= 3.52\times10^{-5}$). A frequency plot of FSS data (Figure S4-7) showed that in the FSS population, the community profiles are very similar to each other which was supported in the Chi^2 test and Spearman rank test. However, in the GS population, there was a small frequency difference for some of the isolates. Chi^2 identified this difference. This concluded that the spatial configuration does not affect the overall distribution but can affect the final frequency of each isolate.

Conclusion

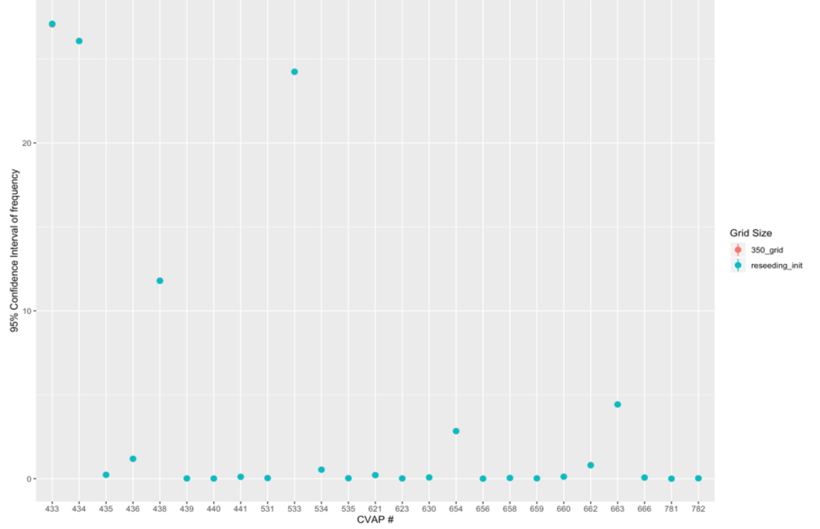

Figure S4-7: The difference of frequency of the consensus and reseeding simulation in FSS simulation. The icon for the reseeded results obscures that of the original.

In conclusion, we developed a cellular automaton in Mathematica allowing us to estimate coexistence of antagonist soil bacterial isolates. Even though many results were published with richness as a measure for coexistence, in the dynamic equilibrium, diversity measures depicting distributions must be included in the argument since distributions, not richness, are independent of grid-size. Spearman Rank is the more appropriate measure to test for significance. Chi^2 is too sensitive unless a $P<0.01$ is used as a critical point. Using Spearman Rank Correlation, no significant differences in distributions were observed when one changes grid size or spatial distributions.

Acknowledgements

We want to thank Katherine Halverson for assistance in developing the cellular automaton.

References

1. Czárán TL, Hoekstra RF, Pagie L. 2002. Chemical warfare between microbes promotes biodiversity. Proc Natl Acad Sci USA 99:786–790.

2. Kerr B, Riley MA, Feldman MW, Bohannan BJM. 2002. Local dispersal promotes biodiversity in a real-life game of rock–paper–scissors. Nature 418:171–174.

3. Kirkup BC, Riley MA. 2004. Antibiotic-mediated antagonism leads to a bacterial game of rock–paper–scissors *in vivo*. Nature 428:412–414.

4. Nahum JR, Harding BN, Kerr B. 2011. Evolution of restraint in a structured rock–paper–scissors community. Proc Natl Acad Sci USA 108:10831–10838.

5. Kerr B. 2007. The Ecological and Evolutionary Dynamics of Model Bacteriocin Communities, p. 111–134. *In* Riley, MA, Chavan, MA (eds.), Bacteriocins. Springer Berlin Heidelberg, Berlin, Heidelberg.

6. Abrudan MI, Smakman F, Grimbergen AJ, Westhoff S, Miller EL, van Wezel GP, Rozen DE. 2015. Socially mediated induction and suppression of antibiosis during bacterial coexistence. Proc Natl Acad Sci USA 112:11054–11059.

7. Zapien-Campos R, Olmedo-Ãlvarez G, Santillan M. 2015. Antagonistic interactions are sufficient to explain self-assemblage of bacterial communities in a homogeneous environment: a computational modeling approach. Front Microbiol 6:489.

8. Rafey A. 2020. Modelling and Simulation of Anaerobic Digestion of Wastewater Sludge using Mathematica. IJERA10:49-54.

9. Gotelli NJ, Colwell RK. Estimating species richness. *In:* Biological Diversity: Frontiers in Measurement and Assessment, Oxford University Press, United Kingdom, p39-54.

10. Gotelli NJ, Chao A. 2013. Measuring and Estimating Species Richness, Species Diversity, and Biotic Similarity from Sampling Data, p. 195–211. *In* Encyclopedia of Biodiversity: Second Edition. Elsevier Ltd.

Appendices SM#4A.

Table S4-Appendix 1A: Spearman rank test result for different grid sizes for the FSS simulation (cell color pink) and the GS simulation (cell color blue)

rho<br>p-value	**Grid size 20**	**Grid size 200**	**Grid size 300**	**Grid size 500**
**Grid size 20**		0.97<br>3.4×10^{-15}	0.995<br>$<2.2\times10^{-16}$	0.995<br>$<2.2\times10^{-16}$
**Grid size 200**	0.95<br>$<2.2\times10^{-16}$		0.97<br>7.6×10^{-7}	0.97<br>7.6×10^{-7}
**Grid size 350**	0.95<br>$<2.2\times10^{-16}$	0.99<br>$<2.2\times10^{-16}$		1.00<br>7.6×10^{-7}
**Grid size 500**	0.95<br>$<2.2\times10^{-16}$	0.99<br>$<2.2\times10^{-16}$	0.999<br>$<2.2\times10^{-16}$	

Table S4-Appendix 1B: Chi^2 test result for different grid sizes for the FSS simulation (cell color pink) and the GS simulation (cell color blue)

Chi^2<br>p-value	**Grid size 20**	**Grid size 200**	**Grid size 350**	**Grid size 500**
**Grid size 20**		550<br>0.25	550<br>0.25	550<br>0.25
**Grid size 200**	1325<br>0.069		600<br>0.24	600<br>0.24
**Grid size 350**	1325<br>0.069	2650<br>0.018		600<br>0.24
**Grid size 500**	1325<br>0.069	2650<br>0.018	2650<br>0.018	

Table S4-Appendix 2: Statistical results of difference between the original and reseeded final distributions.		
	**Chi^2 test**	**Spearman Rank Test**
**FSS simulation**	$Chi^2 = 1921.9$, $P = 0.073$	rho = 0.999, $P = 3.3 \times 10^{-7}$
**GS simulation**	$Chi^2 = 1708.3$, $P = 3.5 \times 10^{-05}$	rho = 1, $P = 3.2 \times 10^{-07}$

Adding data from excel

```
SetDirectory[NotebookDirectory[]];
FSSpath = ToString[NotebookDirectory[]];
FSSdata = Import[FSSpath <> "FSS_1_0.xlsx"];
FSSdata = FSSdata[[1]];
(* the number of isolate in FSS *)
isolates = 25
(* Extracing the interaction matrix data *)
FSSdata = FSSdata[[All, 1 ;; isolates]];
MAXPIXELS = size^2
```

```
Out[∘]= 25

Out[∘]= 400
```

Color

```
NOIsolateCOLOR = White;
(* Assign color to each isolates *)
IsolatesColors = Table[RandomInteger[225, 3] / 225, isolates]
color = Map[RGBColor, IsolatesColors]
```

```
Out[∘]= {{5/9, 8/225, 86/225}, {17/45, 193/225, 8/15}, {7/75, 187/225, 7/9}, {184/225, 107/225, 26/75}, {56/225, 101/225, 134/225}, {124/225, 17/25, 22/25}, {26/75, 13/75, 191/225}, {124/225, 113/225, 0},
 {179/225, 13/25, 92/225}, {31/75, 161/225, 32/75}, {197/225, 3/5, 172/225}, {8/45, 158/225, 18/25}, {16/45, 32/225, 16/45}, {9/25, 2/5, 1/15}, {29/75, 16/25, 8/15}, {49/75, 23/45, 136/225}, {116/225, 4/9, 8/25},
 {71/75, 38/225, 139/225}, {53/75, 3/25, 37/75}, {91/225, 46/225, 124/225}, {187/225, 41/45, 71/75}, {68/225, 188/225, 14/45}, {58/225, 1/25, 112/225}, {22/45, 22/45, 224/225}, {73/225, 38/225, 16/45}}
```

Out[∘]=

Figure S4-Appendix 3: Code for running a simulation.

Module of the simulation

```
fielddayFSS[FSSspecies_] :=
 Module[
  {
   (* padded universe *)
   bigFSS = ArrayPad[FSSspecies, 1, "Periodic"]
  },
  (* will be the list of neighbors of an entry *)

  (* randomly select the focal cell by selecting the row and column randomly which is done size +1 times*)
  (* we did +1 because we had to take the parameters for the bigFSS which has a larger universize than FSSspecies *)

  randomrow = RandomSample[Range[size], size] + 1;
  randomcolumn = RandomSample[Range[size], size] + 1;

  (* use Do function instead of Table because we want to update the FSSspecies universe unlike Table which refers back to the initial FSSspecies
   for ij cells and update the newFSS(another layer which updates separetely from FSSspecies) *)
  Do[

   (* center cell randomly chosen *)

   centerIsolate = bigFSS[[k, l]];
   (* neighbor cell randomly chosen *)
   i = RandomChoice[{k - 1, k, k + 1}];
   j = If[i == k, RandomChoice[{l - 1, l + 1}], RandomChoice[{l - 1, l, l + 1}]];
   neighborIsolate = bigFSS[[i, j]];
   (* first one kills the second *)
   qcenterIsolate = FSSdata[[centerIsolate, neighborIsolate]];
   qneighborIsolate = FSSdata[[neighborIsolate, centerIsolate]];
   (* need to check which q is larger*)
   (* if  qneighborIsolate > qcenterIsolate then take over the cell by the neighbor cell, if qneighborIsolate < qcenterIsolate, no change *)
   If[qcenterIsolate < qneighborIsolate , bigFSS[[k, l]] = neighborIsolate],

   {k, randomrow}, {l, randomcolumn}];
  bigFSS[[2 ;; size + 1, 2 ;; size + 1]]
 ];
```

Figure S4-Appendix 4: Module of the simulation.

Initialize the universe

```
(* randomly assign each isolates to each grid *)
InitialFSS = Table[RandomInteger[{1, isolates}], {i, 1, size}, {j, 1, size}];
(* initialize the universe by assigning Initial FSS to FSS species*)
FSSspecies = InitialFSS;

(* check if the model runs accordingly to the module *)
FSSspecies = InitialFSS;
FSSspecies = fielddayFSS[FSSspecies];
```

Figure S4-Appendix 5: Initializing the universe.

Running grid size 20 for FSS

```
size = 20; (* grid size *)
MAXPIXELS = size ^ 2
(* create a data which looks at the 100th iteration to see the frequncy data for 100 data sets *)
Timing[FrequencyTable20FSS = Table[InitialFSS = Table[RandomInteger[{1, isolates}], {i, 1, size}, {j, 1, size}];
   FSSspecies = InitialFSS; Last[Table[FSSspecies = fielddayFSS[FSSspecies];
      Table[Length[Position[FSSspecies, a]], {a, 1, isolates}], {100}] / MAXPIXELS * 100.], {100}]]
```

Figure S4-Appendix 6: Running the grid size for the 20x20 matrix.

Supplemental Material #4B: Movie: Example of a Probabilistic Cellular Automaton simulation

Movie file “Ogawa et al 2022 Example Probabilistic Cellular Automaton”

This movie is the visualization of a simulation using a 50x50 toroidal regular grid of virtual cells. This grid was randomly seeded resulting in a homologous ethno-sphere. The algorithm randomly chooses the focal cell as well as the neighboring cells and applies the replacement rule as explained in SM#4A. This is followed by 100 iterations and 100 simulations in parallel.

Supplemental Material #5: Molecular identification using 16S sequencing.

Table S5-1 Blast results

		1st published match in NCBI					1st published match in NCBI		
CVAP#	Soil Source	Organism	% identity	Acc#	CVAP#	Soil Source	Organism	% identity	Acc#
433	FSS	*Paraburkholderia*	98.9%	NZ_CP014579.1	401	GS	*Bacillus*	99.7%	NZ_CP051858.
434	FSS	*Paraburkholderia*	98.5%	NZ_CP014579.1	402	GS	*Bacillus*	100.0%	NZ_CP009686.1
435	FSS	*Bacillus*	100.0%	NZ_CP009686.1	403	GS	*Variovorax*	100.0%	NC_014931.1
436	FSS	*Paenibacillus*	94.9%	NZ_CP034346.1	404	GS	*Bacillus*	100.0%	NZ_CP009686.1
438	FSS	*Collimonas*	99.1%	NC_015856.1	406	GS	*Bacillus*	100.0%	NZ_CP009686.1
439	FSS	*Collimonas*	97.7%	NC_015856.1	407	GS	*Bacillus*	100.0%	NZ_CP009686.1
440	FSS	*Bacillus*	99.7%	NZ_CP051858.1	451	GS	*Bacillus*	100.0%	NZ_CP009686.1
531	FSS	*Bacillus*	99.8%	NC_010184.1	453	GS	*Paenibacill*	97.2%	NZ_CP044310.1
534	FSS	*Paenibacillus*	95.2%	NZ_CP034346.1	454	GS	*Bacillus*	100.0%	NZ_CP009686.1
535	FSS	*Bacillus*	100.0%	NC_010184.1	457	GS	*Bacillus*	100.0%	NZ_CP009686.1
621	FSS	*Bacillus*	95.9%	NC_021171.1	458	GS	*Bacillus*	100.0%	NZ_CP009686.1
623	FSS	*Paraburkholderia*	99.6%	NZ_CP014579.1	459	GS	*Paenibacill*	97.5%	NZ_CP044310.1
630	FSS	*Dyella*	96.9%	NZ_CP007444.1	460	GS	*Paenibacill*	97.8%	NZ_CP044310.1
654	FSS	*Collimonas*	98.3%	NC_015856.1	461	GS	*Bacillus*	100.0%	NZ_CP009686.1
656	FSS	*Bacillus*	100.0%	NC_010184.1	462	GS	*Bacillus*	99.8%	NZ_CP007512.1
658	FSS	*Paenibacillus*	92.9%	NZ_CP034346.1	463	GS	*Bacillus*	100.0%	NZ_CP009686.1
659	FSS	*Bacillus*	100.0%	NC_010184.1	464	GS	*Bacillus*	100.0%	NZ_CP009920.1
660	FSS	*Bacillus*	100.0%	NZ_CP009686.1	542	GS	*Lysinibacill*	99.4%	NZ_CP006837.
662	FSS	*Paenibacillus*	94.7%	NC_016935.1	544	GS	*Lysinibacill*	97.8%	NZ_CP010820.1
663	FSS	*Bacillus*	100.0%	NZ_CP009628.1	546	GS	*Bacillus*	100.0%	NZ_CP009686.1
666	FSS	*Paraburkholderia*	98.9%	NZ_CP014579.1	553	GS	*Bacillus*	100.0%	NZ_CP009628.1
781	FSS	*Bacillus*	100.0%	NC_010184.1	559	GS	*Bacillus*	100.0%	NZ_CP009686.1
782	FSS	*Bacillus*	100.0%	NZ_CP051858.1	563	GS	*Bacillus*	100.0%	NZ_CP009686.1
					691	GS	*Bacillus*	100.0%	NZ_CP009686.1
					695	GS	*Bacillus*	100.0%	NZ_CP009686.1
					698	GS	*Bacillus*	100.0%	NZ_CP009686.1
					703	GS	*Bacillus*	100.0%	NZ_CP009628.1
					707	GS	*Bacillus*	100.0%	NZ_CP009369.1
					710	GS	*Bacillus*	100.0%	NZ_CP009628.1
					721	GS	*Bacillus*	100.0%	NZ_CP007512.1
					726	GS	*Bacillus*	100.0%	NZ_CP009628.1
					727	GS	*Bacillus*	100.0%	NZ_CP009628.1
					728	GS	*Bacillus*	100.0%	NZ_CP009628.1
					729	GS	*Bacillus*	100.0%	NZ_CP009686.1
					736	GS	*Bacillus*	100.0%	NZ_CP009628.1
					737	GS	*Bacillus*	100.0%	NZ_CP009628.1
					738	GS	*Bacillus*	100.0%	NZ_CP009628.1
					739	GS	*Bacillus*	100.0%	NZ_CP009628.1
					740	GS	*Bacillus*	100.0%	NZ_CP009628.1
					741	GS	*Bacillus*	100.0%	NZ_CP007512.1
					742	GS	*Bacillus*	100.0%	NZ_CP009686.1
					743	GS	*Bacillus*	100.0%	NZ_CP009628.1
					746	GS	*Bacillus*	100.0%	NZ_CP009628.1
					748	GS	*Bacillus*	99.6%	NZ_CP009351.1
					750	GS	*Bacillus*	100.0%	NZ_CP009351.1
					762	GS	*Bacillus*	100.0%	NZ_CP009351.1
					764	GS	*Bacillus*	97.4%	NZ_CP009600.1
					767	GS	*Bacillus*	100.0%	NZ_CP009628.1

GS means Isolated from Grassland Surface soil

FSS means isolated from Forest SubSurface soil

Yellow means Coverage Not Complete (complete means >1 PCR products, >2 sequence runs, and includes at least 1 forward and 1 reverse sequence determination)

Supplemental Material #6: Contribution of the third component

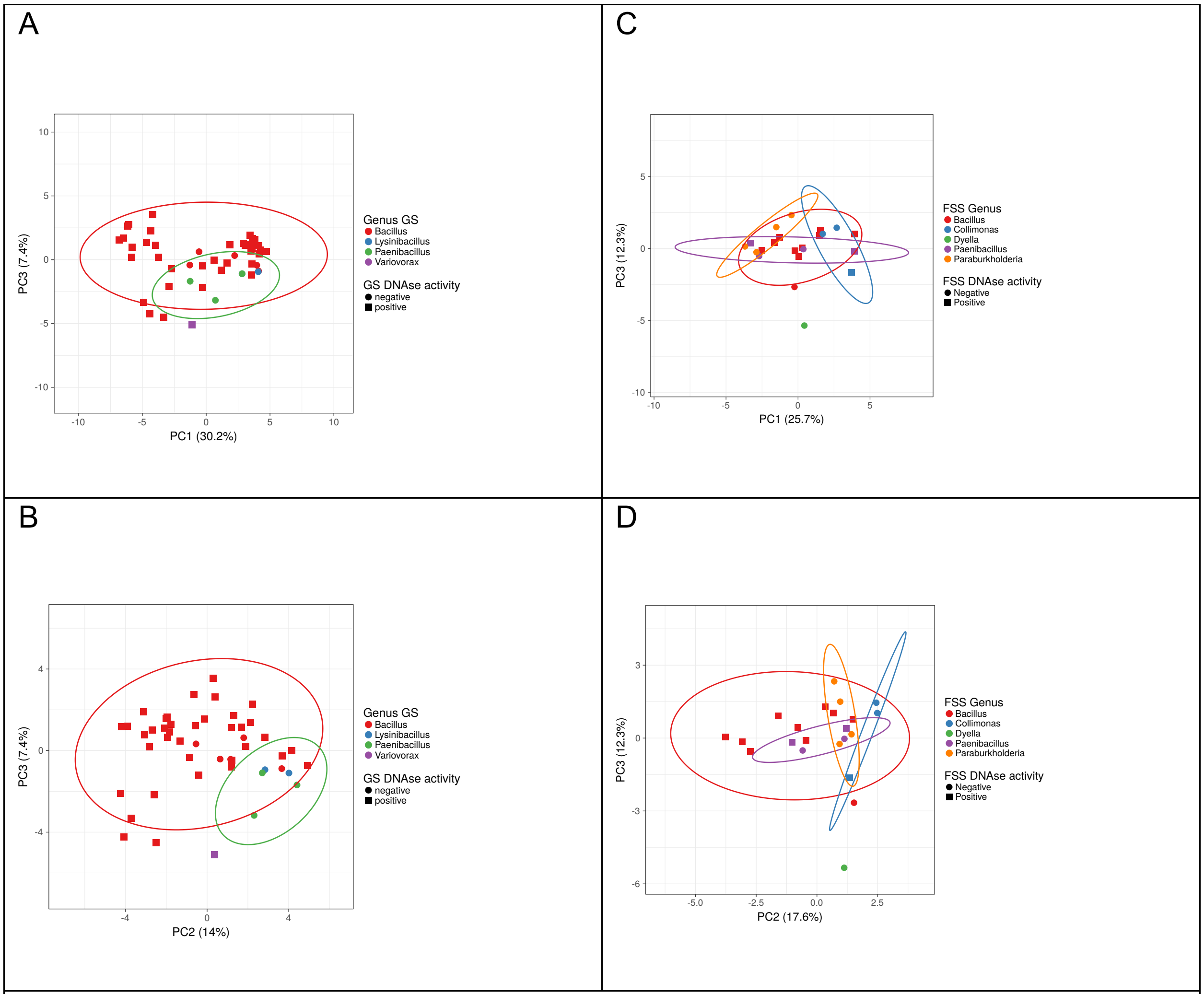

Figure S6-1: Results of the PCA for GS (Panel A, B) and FSS (Panel C, D) for the first three components explaining 51.5% and 55.6% of the variation respectively. With some exceptions, almost all variation in the population is represented mainly by *Bacillus* in both populations. With decreasing contributions of the components, this statement appears less strict.

Reference:

1. Metsalu T, Vilo J. 2015. ClustVis: a web tool for visualizing clustering of multivariate data using Principal Component Analysis and heatmap. Nucleic Acids Res 43:W566–W570.

Supplemental Material#7: Bacterial space and toxin concentrations in GS and FSS soil(s).

Table S#7-1: Estimated bacterial space, distance and relative toxin concentrations in GS and FSS soil(s) (-populations) at the microscale.

			Soil Source	
**Comparison of**	**Note**	**Units**	**GS**	**FSS**
Estimated soil populations	Bacterial load	CFU's/g dry soil	4.41E+06	5.01E+05
	0.53% (GS) and 1.13% (FSS) forms colony. 100%=	cells/g	8.32E+08	4.43E+07
	Expressed in a Volume (Density soil = 1.3g/ml (1))	cells/ml	1.08E+09	5.76E+07
	Average space around cell	µL/cell	9.24E-07	1.73E-05
	Average distance between cells (=2xr), r=Radius	2r in µm	12.1	32.1
	Volume Sphere of influence ($V=4/3*\pi*r^3$)	μm^3	9.24E+02	1.73E+04
	Fold difference in volume relative to GS			18.8
Estimated # Antagonists	Antagonists (%)		10.3	5.4
	# Antagonists/g dry soil	CFU's/g dry soil	4.54E+05	2.71E+04
	0.53% (GS) and 1.13% (FSS) forms colony. 100%=	cells/g	8.57E+07	2.39E+06
	Expressed in a Volume (Density soil = 1.3g/ml (1))	cells/ml	1.11E+08	3.11E+06
	Average space per antagonist	µL/cell	8.98E-06	3.21E-04
	Average distance between antagonists (=2xr), r=Radius	2r in µm	25.8	85.0
	Volume Sphere of influence ($V=4/3*\pi*r^3$)	μm^3	8.98E+03	3.21E+05
	Fold difference in volume relative to GS			35.8
Corrected for Connectance	Antagonists (%)	%	10.3	5.4
	Connectance (%)	%	41.2	30.6
	Effective Antagonists (=Antagonists(%)xConnectance (%)x100)	$\%^2$	4.2	1.7
	Effective Antagonists	CFU's/g dry soil	1.87E+05	8.28E+03
	0.53% (GS) and 1.13% (FSS) forms colony. 100%=	cells/g dry soil	3.53E+07	7.33E+05
	Expressed in a Volume (Density soil = 1.3g/ml (1))	cells/ml	4.59E+07	9.52E+05
	Average space per Effective Antagonists	µL/cell	2.18E-05	1.05E-03
	Average distance between Effective Antagonists (=2xr), r=Radius	2r in µm	34.7	126.1
	Volume Sphere of influence ($V=4/3*\pi*r^3$)	μm^3	2.18E+04	1.05E+06
	Fold difference in volume relative to GS			48.2

Reference:

1. Raynaud X, Nunan N. 2014. Spatial Ecology of Bacteria at the Microscale in Soil. PLoS ONE 9:e87217.

Supplemental Material #8: ExDNase activity strain *Serratia* sp. CWZ222

Results

To test for antagonist activity towards *Staphylococcus sp.* CWZ226 (1) and for DNase activity (2), DNA-plates (Difco cat#263220) were seeded with *Staphylococcus sp.* CWZ226 as described in the material and methods, and soil isolates were tested for their ability to inhibit *Staphylococcus* sp. CWZ226 and for exDNase activity. The positive control for exDNase activity is *Serratia* sp. CWZ222 as determined by 16S sequencing. The positive control for inhibition of *Staphylococcus* sp. CWZ226 is strain *Pseudomonas* sp. CVAP#3 (1). The negative control was *Staphylococcus* sp. CWZ226, which should not produce a ZOI, nor harbor exDNase activity. Plates were incubated for three days at 25˚C and the Zone of Inhibition (ZOI) was determined as well as the exDNase activity. The results depicted in Figure S8-1 shows that *Serratia* sp. CWZ222 does not inhibit *Staphylococcus* sp. CWZ226, but does hydrolyze DNA. As expected, *Staphylococcus* sp. CWZ226, did not show inhibition of itself, did not produce DNases. *Pseudomonas* sp. CVAP#3 does inhibit *Staphylococcus* sp. CWZ226, however does not show exDNase activity.

A

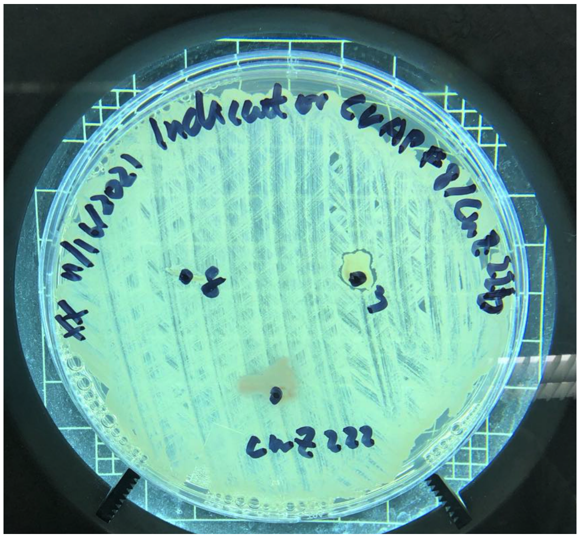

B

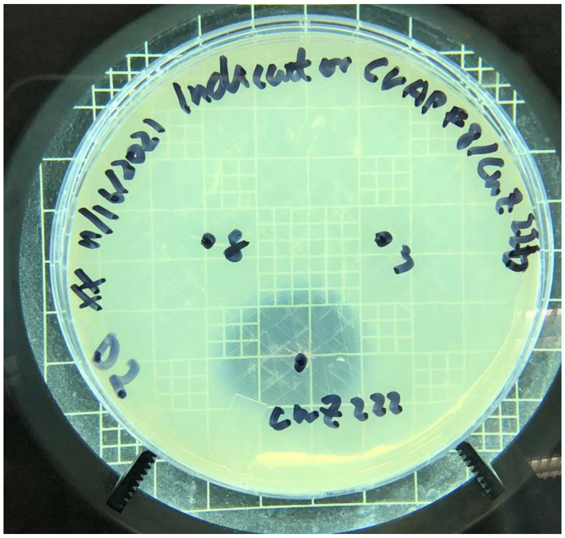

Figure S8-1: Antagonist (Insert A) and DNase activity (Insert B) on DNA plates with indicator *Staphylococcus* sp. CWZ226. Tested are: *Staphylococcus* sp. CWZ226 (Indicated with #8, Negative for antagonism and negative for DNase activity), *Pseudomonas* sp. CVAP#3 (Indicated with #3, Positive for antagonism and negative for DNase activity), and *Serratia* sp. CWZ222 (Indicated with CWZ222, Negative for antagonism and positive for exDNase activity).

References:

1. McCall BL, Vriezen JAC. 2021. Draft Genome Sequences of *Staphylococcus* sp. Strain CWZ226, of Unknown Origin, and *Pseudomonas* sp. Strain CVAP#3, Antagonistic to Strain CWZ226. Microbiol Resour Announc 10:e00688-21.

2. Andrade JP, de Souza HG, Ferreira LC, Cnockaert M, De Canck E, Wieme AD, Peeters C, Gross E, De Souza JT, Marbach PAS, Góes-Neto A, Vandamme P. 2021. *Burkholderia perseverans* sp. nov., a bacterium isolated from the Restinga ecosystem, is a producer of volatile and diffusible compounds that inhibit plant pathogens. Braz J Microbiol 52:2145–2152.
